# Differentially expressed microRNAs in diapausing versus HCl-treated *Bombyx* embryos

**DOI:** 10.1371/journal.pone.0180085

**Published:** 2017-07-11

**Authors:** Wentao Fan, Yangsheng Zhong, Mingyue Qin, Bimin Lin, Fangyan Chen, Huichao Yan, Wenchu Li, Jianrong Lin

**Affiliations:** Department of sericulture, College of Animal Science, South China Agricultural University, Guangzhou, Guangdong, China; Kunming University of Science and Technology, CHINA

## Abstract

Differentially expressed microRNAs were detected to explore the molecular mechanisms of diapause termination. The total small RNA of diapause-destined silkworm eggs and HCl-treated eggs was extracted and then sequenced using HiSeq high-throughput method. 44 novel miRNAs were discovered. Compared to those in the diapause-destined eggs, 61 miRNAs showed significant changes in the acid-treated eggs, with 23 being up-regulated and 38 being down-regulated. The potential target genes of differentially expressed miRNAs were predicted by miRanda. Gene Ontology and KEGG pathway enrichment analysis of these potential target genes revealed that they were mainly located within cells and organelles, involved in cellular and metabolic processes, and participated in protein production, processing and transportation. Two differentially expressed genes, *Bombyx mori SDH* and *Bmo-miR-2761-3p*, were further analyzed with qRT-PCR. *BmSDH* was significantly up-regulated in the HCl-treated eggs, while *Bmo-miR-2761-3p* was down-regulated. These results suggested that these two genes were well coordinated in silkworm eggs. Dual luciferase reporter assay demonstrated that *Bmo-miR-2761-3p* inhibited the expression of *BmSDH*.

## Introduction

Diapause is an important physiological mechanism for insects to adapt to harsh environmental conditions [1]. At 25°C, silkworm enters diapause at its early embryo stage 24h after oviposition [2]. A significant drop in oxygen consumption and the conversion of glycogen to sorbitol and glycerol slow down division of embryonic cells and completely stops the cells in G2 stage of the cell cycle 3 days after oviposition [3]. Diapause can be prevented if diapause-destined eggs are artificially treated with HCl. Many mechanisms for silkworm diapause have been reported. For example a key enzyme sorbitol dehydrogenase (SDH) utilizes sorbitol to synthesize glycogen to provide energy [4]. Its well known that SDH is a molecular marker for *Bombyx* embryo development. The activity of SDH can’t be detected in diapause-destined eggs but is measurably increased in HCl-treated eggs 2 days after oviposition [5,6]. NAD/NADH and NADP/NADPH can regulate respiratory chain and provide energy during silkworm embryonic development [7]. Some researchers believe that the lack of *BmCyclin* B/B3 was the cause of cell cycle arrest during diapause [8]. As widely known that the continuous division and proliferation of embryonic cells is the key to break silkworm diapause, however,the molecular mechanisms of diapause is unclear. With the next-generation sequencing technology, researchers began to use high-throughput screening approach to determine the differentially expressed genes of diapause and non-diapause eggs on the transcriptomics [9,10] and proteomic levels[11,12] to study *bombyx* embryonic development.

MicroRNAs (miRNAs) are a class of endogenously initiated non-coding RNAs (ncRNAs) about 21–25 nucleotides in length, They widely exist in animals, plants, nematodes, viruses, and other organisms, and play an important role in various physiological processes [13,14]. Two miRNAs, lin 4 [15] and let 7 [16] have been found to regulate embryo development. Extensive regulation functions of miRNAs have been continually discovered in recent years and researches showed that miRNAs could regulate cell multiplication, differentiation, apoptosis [17,18], embryo development [19] and hormone secretion[20].

Silkworm is a model organism of lepidoptera insects, therefore, studying silkworm miRNA is important to explain the molecular mechanisms of insects. Previous studies on silkworm miRNA expression patterns at different developmental stages have shown that 248 miRNAs are specifically expressed in the egg or pupa stage [21], and 14 novel stage-specific miRNAs are expressed from the 4th instar stage to adult stage [22].Furthermore, *Bmo-miR-2763* regulated diapause initiation via diapause hormone receptors. *Bmo-miR-2733e* was believed to target NADPH-cytochrome P450 reductase, which is important for the synthesis of 20-hydroxylation ecdysone in insect embryonic development [23]. So far, 487 pri-miRNA precursors and 567 mature miRNAs of silkworm have been registered in miRBase (http://www.mirbase.org/).

In the present study, day-3 eggs of bivoltine 932 strain silkworm were treated with HCl. The Small RNA (sRNA) libraries of diapause-destined eggs and HCl-treated eggs were constructed respectively. High-throughput sequencing with HiSeq technology was used to compare the differentially expressed miRNAs in two types of silkworm eggs. GO classification and KEGG pathway enrichment analysis of candidate target genes were used to detect the expression of *Bmo-miR-2761-3p* and *Bombyx mori* sorbitol dehydrogenase(*BmSDH*). *Bmo-miR-2761-3p* was identified as an inhibitor of *BmSDH* expression using (Dual luciferase reporter assay) DLR assay. These differentially expressed miRNAs might provide the reference for further study of the molecular mechanisms of diapause.

## Materials and methods

### Materials

Day-3 diapause-destined eggs from 932 strain silkworms *Bombyx mori (B*.*mori*), were treated with HCl acid (specific gravity of 1.075 kg/L) at 116 ℉ (46.7°C) for 13.5 min. After acid treatment, eggs were thoroughly washed in running water for 30 min to remove traces of acid, and were air-dried. The diapause-destined eggs without acid treatment were used as controls. HCl-treated eggs and control eggs were stored at 25°C with 80% relative humidity. Diapause termination was confirmed by 95%+ hatchability of HCl-treated eggs. A few non-treated eggs were taken from each group. Non-diapause or unfertilized eggs were distinguished from diapause-destined eggs.

0.1g samples were taken from control eggs and HCl-treated eggs respectively daily from Day 3 to 7. All samples were stored at -80°C for further use. Three duplicates were obtained from each group.

The Mammalian HEK293T cell line was maintained in DMEM medium (Dingguo, Beijin, China) with high glucose and 10% fetal bovine serum (Dingguo) at 37°C and under 5% CO2.

### RNA isolation and sRNA library construction, and sequencing

Total RNA of control eggs and HCl-treated eggs was extracted 1 day after HCl treatment (Day 4 eggs). Quality of the extracted RNA was determined by electrophoresis on 2.0% agarose gel before the RNA was used to construct cDNA library using NEBNext Multiplex Small RNA Sample Prep Kit (Illumina). Following manufacturer’s recommendations and index codes were added to attribute sequences to each sample. Briefly, NEB 3’ SR Adaptor was directly, and specifically ligated to 3’ end of miRNAs, siRNA and piRNA. After the 3’ ligation reaction, the SR RT Primer hybridized to the excess of 3’ SR Adaptor (that remained free after the 3’ ligation reaction) and transformed the single-stranded DNA adaptor into a double-stranded DNA molecule. This step is important to prevent adaptor-dimer formation, besides, dsDNAs are not substrates for ligation mediated by T4 RNA Ligase 1 and therefore do not ligate to the 5’ SR Adaptor in the subsequent ligation step. 5’ ends adapter was ligated to 5’ ends of miRNAs, siRNA and piRNA. Then first strand cDNA was synthesized using M-MuLV Reverse Transcriptase (RNase H^–^). PCR amplification was performed using LongAmp Taq 2X Master Mix, SR Primer for illumina and index (X) primer. PCR products were purified on a 8% polyacrylamide gel (100V, 80 min). DNA fragments corresponding to 140~160 bp (the length of small noncoding RNA plus the 3’ and 5’ adaptors) were recovered and dissolved in 8 μL elution buffer. At last, library quality was assessed on the Agilent Bioanalyzer 2100 system using DNA High Sensitivity Chips.

The clustering of the index-coded samples was performed on a cBot Cluster Generation System using TruSeq SR Cluster Kit v3-cBot-HS (Illumia) according to the manufacturer’s instructions. After cluster generation, the library preparations were sequenced on an Illumina Hiseq 2000 platform[24] and 50 bp single-end reads were generated.And the rawdata were upload to the GEO database, the accession numbers is GSE95576.

Raw data (raw reads) of fastq format were firstly processed through custom perl and python scripts. In this step, clean data (clean reads) were obtained by removing reads containing ploy-N, with 5’ adapter contaminants, without 3’ adapter or the insert tag, containing ploy A or T or G or C and low quality reads from raw data. At the same time, Q20, Q30, and GC-content of the raw datas were calculated. Then, chose a certain range of length from clean reads to do all the downstream analyses.

### miRNA identification

sRNAs of 18–40 nt length were selected and located on the silkworm chromosome through bowtie (http://bowtie-bio.sourceforge.net/index.shtml). The density statistics of the mapped reading on silkworm chromosome was obtained and sRNAs were aligned with known miRNAs using information on sequence, length, and secondary structure using miRBase. Software miREvo: an integrative microRNA evolutionary [25] and miRdeep2 [26] were integrated to predict novel miRNAs through exploring the secondary structure (Figs A-Rr in S1 File), the Dicer cleavage site and the minimum free energy of the small RNA tags unannotated in the former steps[27]. At the same time, custom scripts were used to obtain the identified miRNA counts as well as base bias on the first position with certain length and on each position of all identified miRNAs respectively.

### Screening of differentially expressed miRNAs

Reads count data were normalized by transcripts per million (TPM) [28], TPM = reads count * 10^6 / Total mapped reads. The differences in miRNA expression were analyzed by DEGseq [29]. By the DEGseq nbinomTestGLM function it return the p value for the statistical significance of the the folds change, and the qvalue was adjusted p value by Benjamini-Hochberg procedure, which controls false discovery rate.To limit false positive rate, all samples were screened with parameter setting of qvalue < 0.01 and |log_2_ (fold change)| >1.

### Prediction and analysis of target genes

The target genes from differentially expressed (DE) miRNAs were predicted by miRanda [24]. Each set of DE miRNAs were designed as candidate target (CT) genes which were further analyzed based on GO (Gene Ontology annotation, http://wego.genomics.org.cn/;http://geneontology.org/) and KEGG (Kyoto Encyclopedia of Genes and Genomes, http://www.genome.jp/kegg/) pathway enrichment. Drosophila melanogaster genes used as background.

### Quantitative real-time PCR (qRT-PCR)

Total RNA of control eggs and HCl-treated eggs (day 3 to 7 old eggs) was extracted according to the manufacturer’s instructions (Sangon, Shanghai, China). AMV First Strand cDNA Synthesis Kit (Sangon) was applied to synthesize first strand cDNA. Random Primer p(dN)6 was used to construct mRNA cDNA and Stem loop (SL) primers were used to synthesize miRNAs cDNA (Table 1). The first strand and specific cDNAs were used as templates in the amplification of *BmSDH* (gb|DQ443393.1) and *Bmo-miR-2761-3p* (MIMAT0013635). Briefly, PCR reaction was performed as follows: 10 μL of 2X ABI SybrGreen PCR Master Mix, 1 μL of 10 μM primer F, 1 μL of 10 μM primer R, 6 μL of ddH_2_O, and 2 μL of cDNA template were mixed at 95°C for 3 min, followed by 40 cycles at 95°C for 15 s and 60°C for 40 s. *BmActin A3* and *U6* were used as internal control genes for *BmSDH* and *Bmo-miR-2761-3p*, respectively.

**Table 1:** Primers used in this study. SL (Stem loop) primers were used for cDNA synthesis of miRNAs.

Primer name	Primer sequence(5’-3’)
*RNA 5’ Adapter*	GTTCAGAGTTCTACAGTCCGACGATC
*RNA 3’ Adapter*	AGATCGGAAGAGCACACGTCT
*Bmo-miR-2761-3p-F SL primer*	CTCAACTGGTGTCGTGGAGTCGGCAATTCAGTTGAGTCCATCGA
*Bmo-miR-2761-3p-F*	ACACTCCAGCTGGGTGTGTGGAACCGTCG
*Bmo-miR-2761-3p-R*	TGGTGTCGTGGAGTCG
*BmSDH-F*	GCCTCCTTACCATGCTCACA
*BmSDH-R*	CGAAATCCAAACGGCTCTG
*BmActin A3-F*	TTGGTAGTAGACAATGGCTCCG
*BmActin A3-R*	ACCTCTTTTGCTCTGTGCCTC
*U6-F*	AAAGGCAGCCAAGTAACAGGA
*U6-R*	AGCACAAGGTTTAGAAGGGGAT
*BmSDH 3’UTR-F*	AAACGAGCTCATGGTTCCTCAAAAACACATGC
*BmSDH 3’UTR-R*	CTAGTCTAGAATTTCTGCTAGAAAACTGGTATCG
*PC-F*	CTCCATCGACGACGGTTCCACACATCCATCGACGACGGTTCCACACAT
*PC-R*	CTAGATGTGTGGAACCGTCGTCGATGGATGTGTGGAACCGTCGTCGATGGAGAGCT

Note: dotted line bases represents SacI and XbaI restriction enzyme cutting sites,solid line bases represents the gene or miRNA sequence.

### Dual luciferase reporter (DLR) assay

*Bmo-miR-2761-3p* mimics and negative control (NC) mimics were purchased from Dingguo (Beijing, China). Dual luciferase expression vector pmirGlo (Promega, S1 Fig) includes firefly luciferase (*Luc2*) and Renilla luciferase *(hRluc-neo fusion*). *BmSDH* 3’UTR (746 bp) was cloned and inserted into the *Luc2* 3’UTR to construct wild type (WT) plasmid, while *Bmo-miR-2761-3p* inhibitor was inserted into *Luc2* 3’UTR to construct positive control (PC) plasmid. Restriction enzyme cutting sites were SacI and XbaI.

HEK293T cell line was used for DLR assay. Co-transfection was classified into two groups: PC group, including PC + NC mimics and PC + *Bmo-miR-2761-3p* mimics, and WT group, including WT + NC mimics and WT + *Bmo-miR-2761-3p* mimics. Cells in each well were transfected with 500 ng reporter plasmid and 15 pM mimics. The DLR Assay (Promega) was performed according to the manufacturer’s protocol 24 h after the transfection.

## Results

### Identification sRNA of silkworm eggs

**Fig 1:**
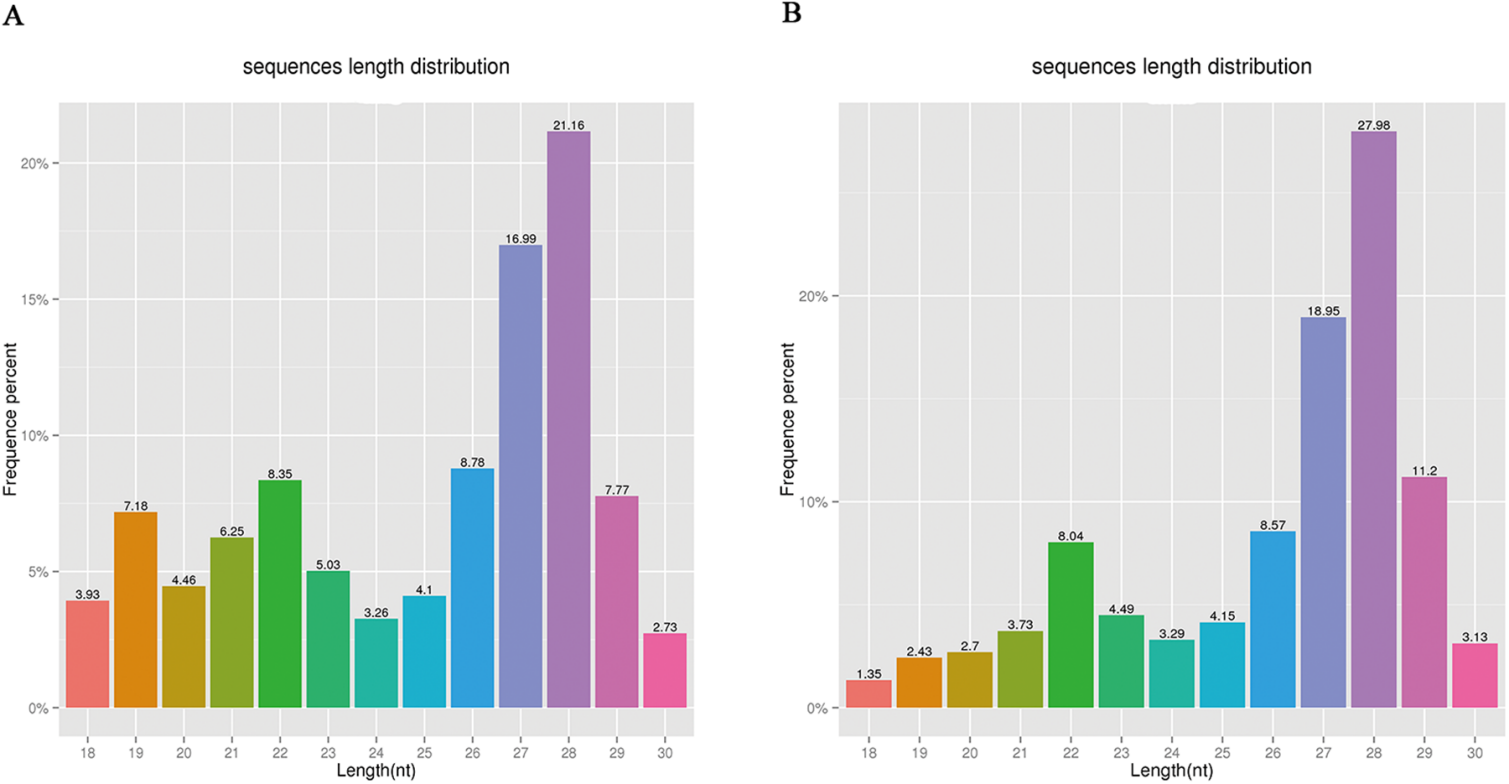
Length distribution of sRNA. (A) Length distribution of sRNA in control eggs, (B) Length distribution of sRNA in HCl-treated eggs. Y asix represents the precentage of special sRNA in total, X axis represents length of sRNA.

**Fig 2:**
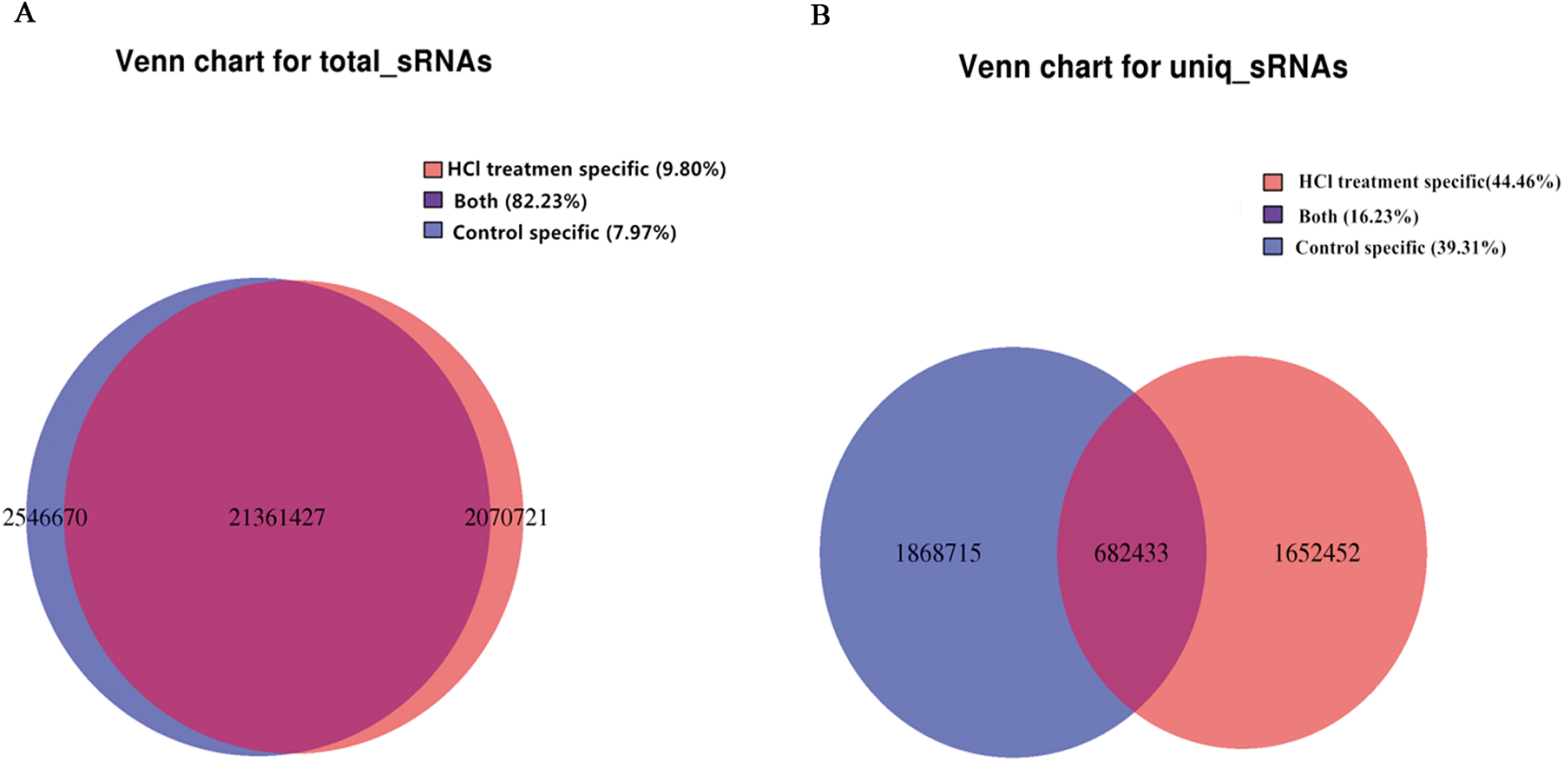
Venn chart for sRNA. (A) Venn chart for sRNA of total sRNA, (B) Venn chart for sRNA of unique sRNA.

**Fig 3:**
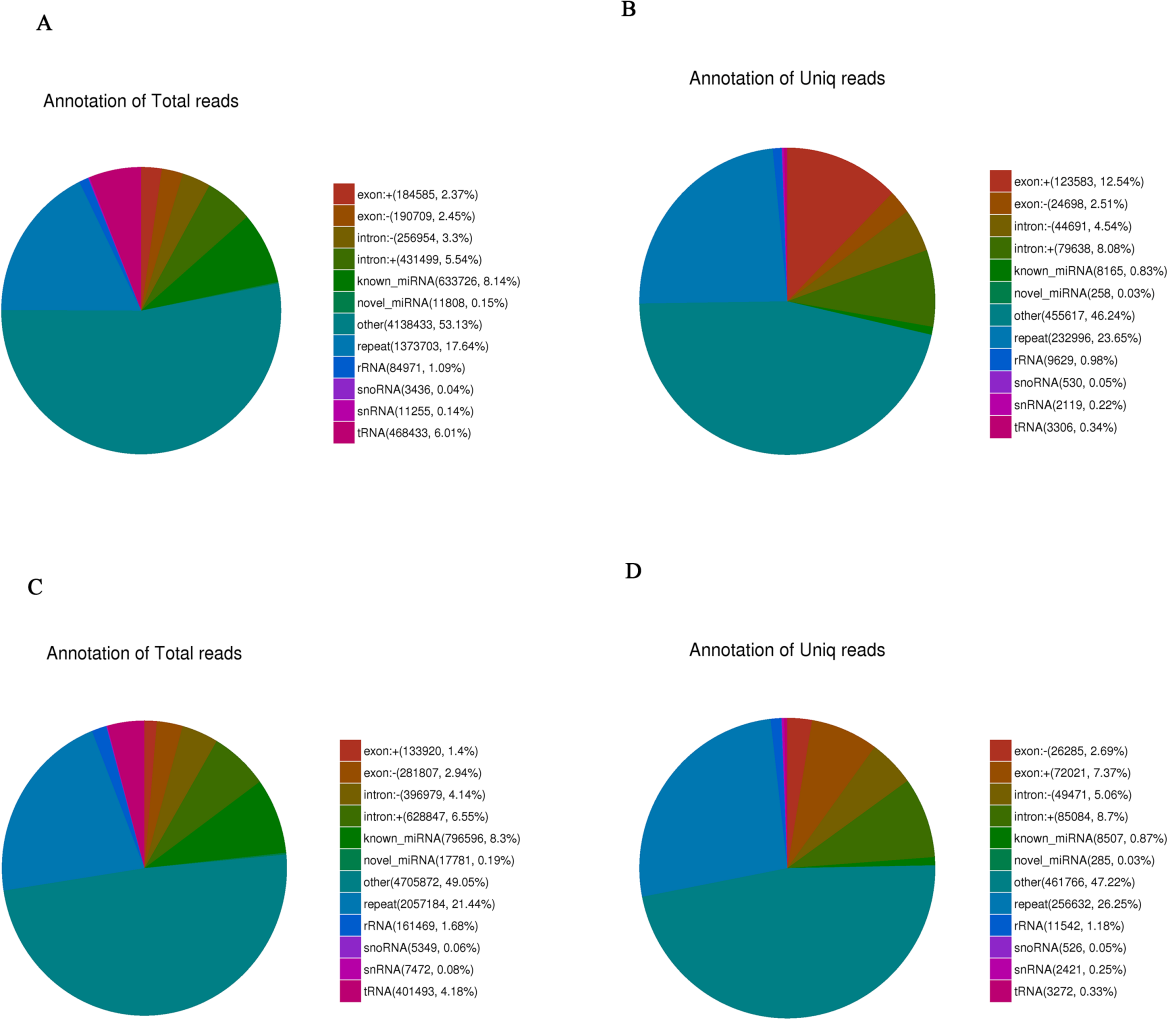
Distribution of ncRNA. (A) ncRNA distribution of total sRNA in control eggs, (B) ncRNA distribution of unique sRNA in control eggs, (C) ncRNA distribution of total sRNA in HCl-treated eggs, (D) ncRNA distribution of unique sRNA in HCl-treated eggs.

sRNA was isolated from control eggs and HCl-treated eggs on day-4 post-oviposition. HCl-treated eggs showed 17049696 clean reads, while 17309699 clean reads were detected in control eggs. 9594769 and 7789512 clean reads mapped to silkworm chromosomes respectively (S1 Table). sRNA lengths were in the range of 18 to 30 nt (Fig 1). 7.79% and 9.80% of total sRNA were specific sRNA in the control eggs and HCl-treated eggs, respectively (Fig 2A). 39.31% and 44.46% were unique sRNA, respectively (Fig 2B). 8.29% and 8.49% sRNAs were miRNAs for the control group and HCl-treated group, respectively (Fig 3A and 3C). For further research, the mapped reads were analyzed and sRNA was compared with repeated sequence, exon, and intron, 44 novel miRNAs were discovered (S2 Table).

### Different miRNAs expressions

Statistic analysis showed that there were 61 miRNAs with significant expression changes, including 51 known and 10 novel miRNAs. Among those 61 differentially expressed (DE) miRNAs, 23 were up-regulated and 38 were down-regulated (S3 and S4 Tables). Some specifically expressed miRNAs may be associated with silkworm diapause. Herein, *novel_45* and *Bmo-miR-2809* were only found in the HCl-treated eggs while *Bmo-miR-3245/3228/3274/novel_48* disappeared after HCl treatment. In addition, *Bmo-miR-2733c/3384-3p/ 6497-3p/6498-3p/306a-5p* expression levels were much higher than other DE miRNAs (Table 2). 17 up-regulated miRNAs and 27 down-regulated miRNAs had a fold change of 1 to 3. Four up-regulated miRNAs and 9 down-regulated miRNAs had a 3 to 5 fold change. Three up-regulated miRNAs and 2 down-regulated miRNAs had a >5 fold change.

**Table 2:** miRNAs with specific expression.

miRNA	HCl-treated_TPM	Control_TPM	fold change	q-value
*novel_45*	19.115	0.000	5.257	1.56E-05
*Bmo-miR-2809*	11.150	0.000	4.479	0.0011882
*Bmo-miR-3245*	0.000	28.295	-5.823	2.68E-08
*novel_48*	0.000	16.977	-5.086	1.70E-05
*Bmo-miR-3228*	0.000	7.545	-3.916	0.0051246
*Bmo-miR-3274*	0.000	7.545	-3.916	0.0051246
*Bmo-miR-2733c*	100505.148	39512.512	1.347	0
*Bmo-miR-3384-3p*	140.177	1829.720	-3.706	0
*Bmo-miR-6497-3p*	13014.138	37712.974	-1.535	0
*Bmo-miR-6498-3p*	684.955	1582.613	-1.208	9.96E-102
*Bmo-miR-306a-5p*	6260.167	12642.042	-1.014	0
*Bmo-miR-2842*	331.327	1.886	7.457	6.30E-71
*Bmo-let-7-5p*	47.788	20.749	1.204	0.0036874

Note: fold change = log2 (HCl-treated_TPM / Control_TPM), positive value represents up-regulation, negative value represents down-regulation, TPM represents transcripts per million, q-value closer to 0 represents more significant difference.

### Target prediction and analysis

**Fig 4:**
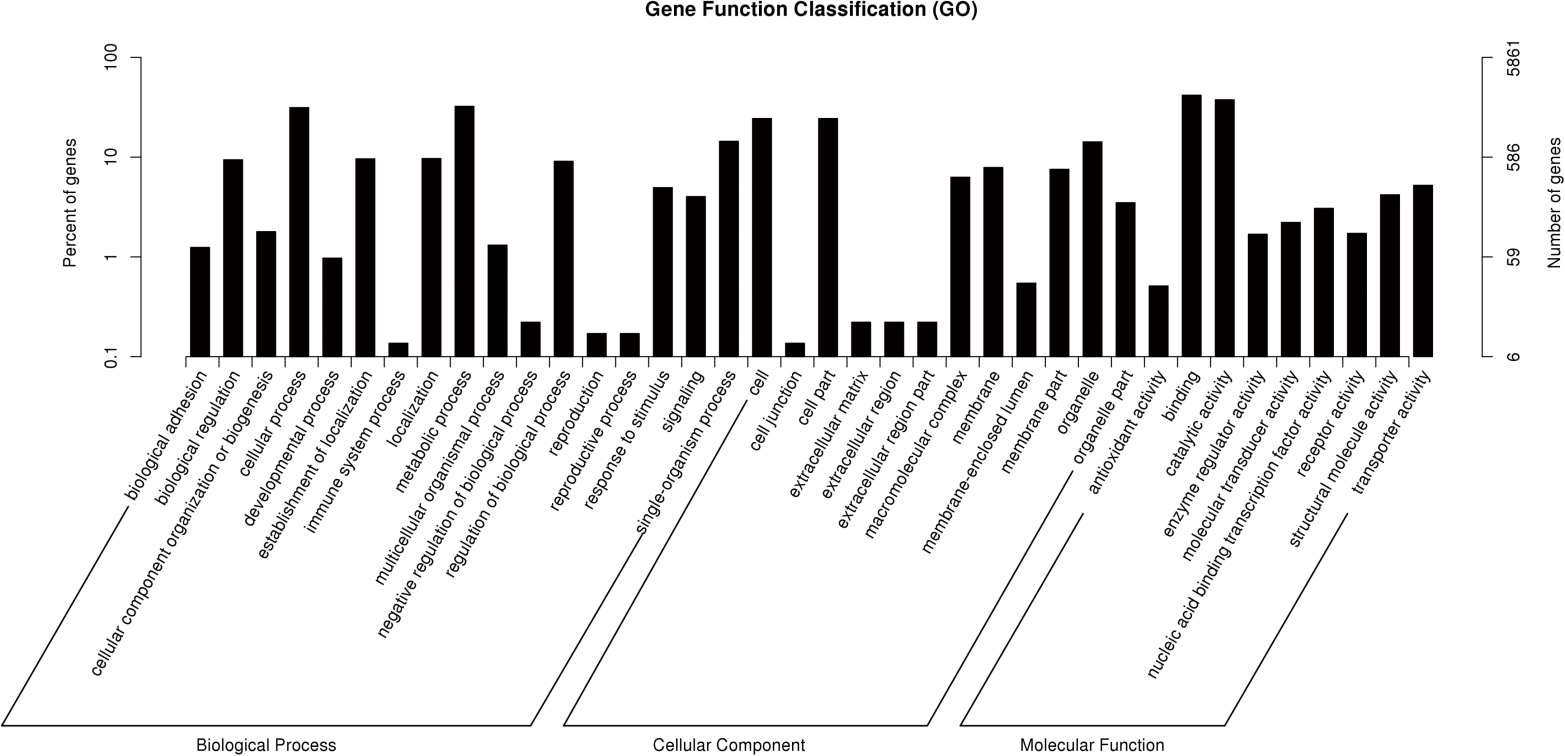
Gene ontology annotation of candidate target genes predicted by miRanda. Candidate target genes were classified into three categories: Biological Process, Cellular Component, and Molecular Function.

**Fig 5:**
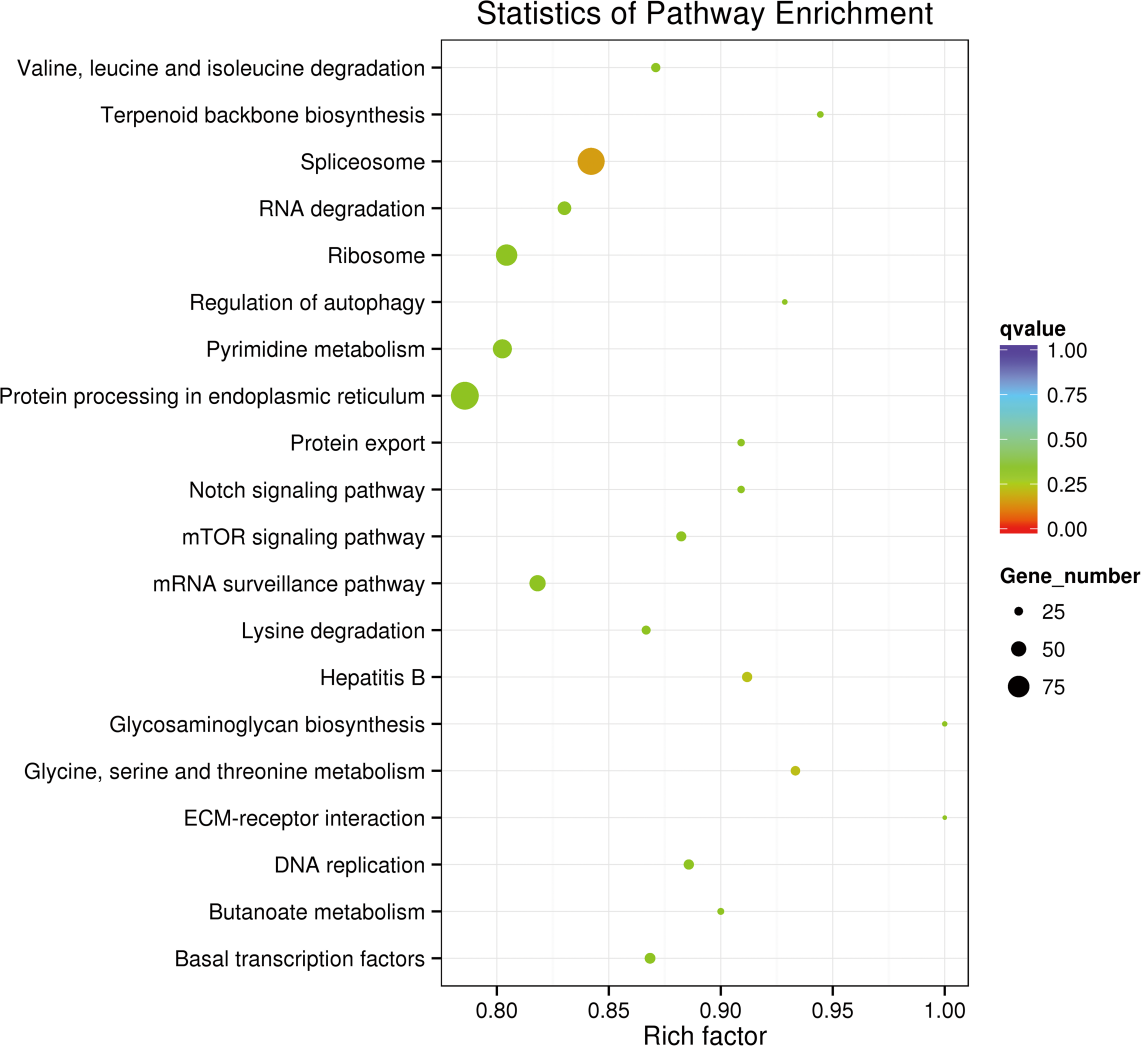
KEGG pathways mapped based on miRNAs candidate target genes. Y axis represents KEGG pathway, X axis Rich factor represents the percentage of Candidate target genes enriched in pathway, the qvalue closer to 0 represents enriched more significant.

To further analyze DE miRNAs in silkworm diapause, GO annotation and KEGG pathway enrichment of candidate target (CT) genes were performed. Drosophila melanogaster genes used as background.The GO annotation suggested that CT genes were mainly involved in cellular and metabolic processes, single-organism process and biological regulation. In Cellular component class suggested CT genes were components of cells, organelles and biological membranes where the locale of protein synthesis and transport processes. In terms of molecular functions, CT genes might function in combining, catalytic, nucleic acid binding transcription factor, suggesting the miRNAs might affect embryo development through regulating enzyme activity (Fig 4). Moreover, CT genes were significant enriched in 18 KEGG pathways (Fig 5). The pathway for protein processing in endoplasmic reticulum had most rich target genes (99), with Spliceosome at the second place (96) and Ribosome at the third (74). These three pathways are the main ones for protein synthesis, process and modification. In addition, CT genes also participate in the regulation of cell cycle, such as mTOR, Notch, Gly, and Ser/Thr metabolism. The pathway with least target genes (9) regulates ECM-receptor interaction.

### Expressions pattern of *Bmo-miR-2761-3p* and *BmSDH*

**Fig 6:**
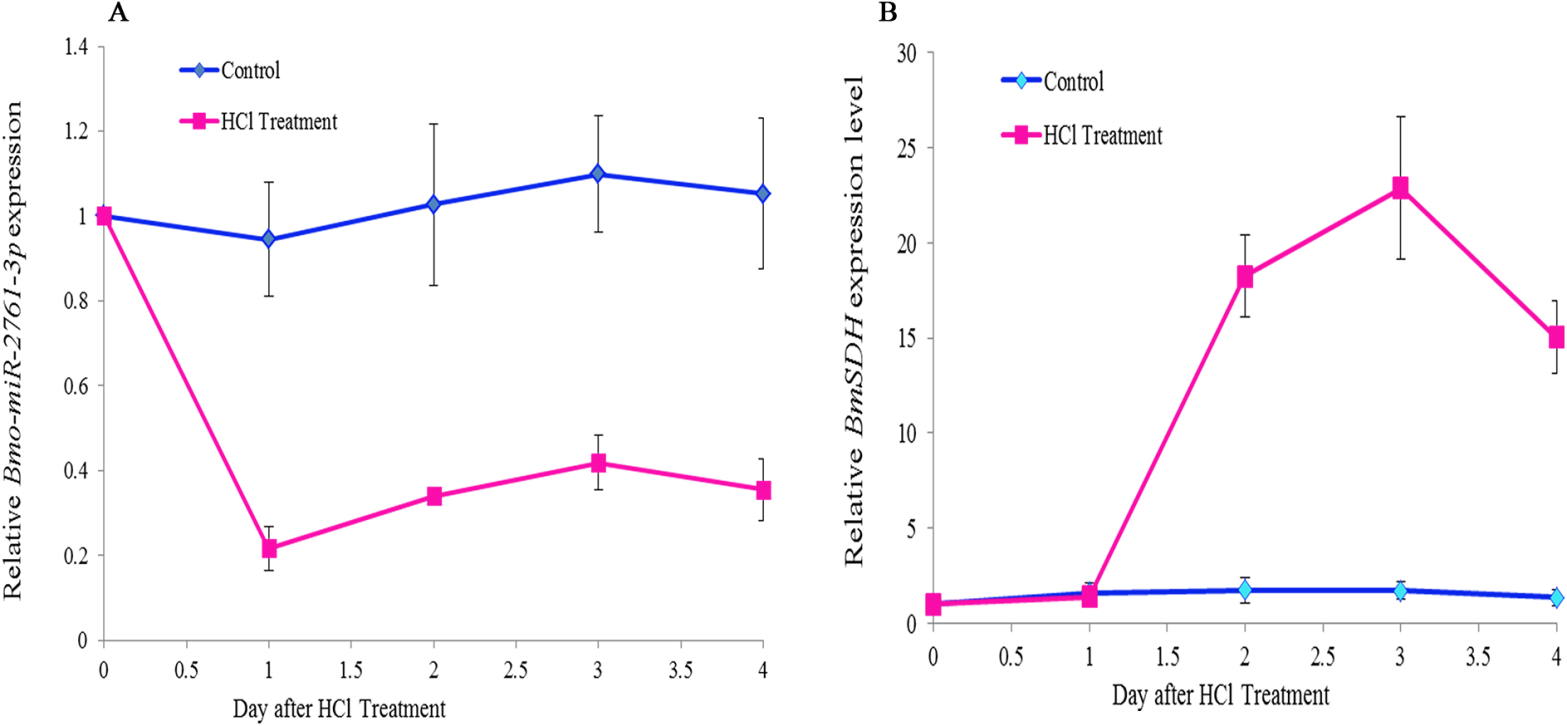
Expressions pattern of *Bmo-miR-2761-3p* and *BmSDH* from 3 to 7 day old eggs of silkworm, *B*. *mori*. Each time point was replicated three times using independently collected samples. Error bar = 1 SD. (A) Relative *Bmo-miR-2761-3p* expression level; (B) Relative *BmSDH* expression level.

Since the *BmSDH* gene play an important role in the *B*.*mori* diapause, we interesting in which miRNAs would regulated its expression. In the present study, to investigate the regulatory function in silkworm diapause termination, used the DE miRNAs as candidate, by the target prediction, we found only *Bmo-miR-2761-3p* have target position in the *BmSDH* 3’UTR. To confirm whether there is a good correlation in the transcriptional level between them, qRT-PCR was used to detect. By the qRT-PCR screening in day 3 to 7 old eggs, however, *Bmo-miR-2761-3p* and *BmSDH* expressed differently and have a strong negative correlation. Fig 6 showed that the two genes were relatively stable in control eggs. However, compared to the control eggs, the relative *Bmo-miR-2761-3p* transcript level in HCl-treated eggs was only 0.220, a 76.01% down-regulation 1 day after HCl treatment. The expression of *Bmo-miR-2761-3p* rose slightly afterwards, but maintained at a relatively low level (Fig 6A). However, *BmSDH* was up-regulated slightly in the HCl-treated eggs. It rose sharply 3 days later, and the relative transcription level was up to 43.87 folds before it returned to base level (Fig 6B). Student t-test analysis showed that the gene expression was significantly different between HCl-treated eggs and control eggs for both *Bmo-miR-2761-3p* and *BmSDH* (p<0.01). The up-regulation of *BmSDH* was closely associated with the down-regulation of *Bmo-miR-2761-3p* (R^2^ = 0.96).

### *Bmo-miR-2761-3p* down-regulated the expression of *BmSDH* gene in vitro

**Fig 7:**
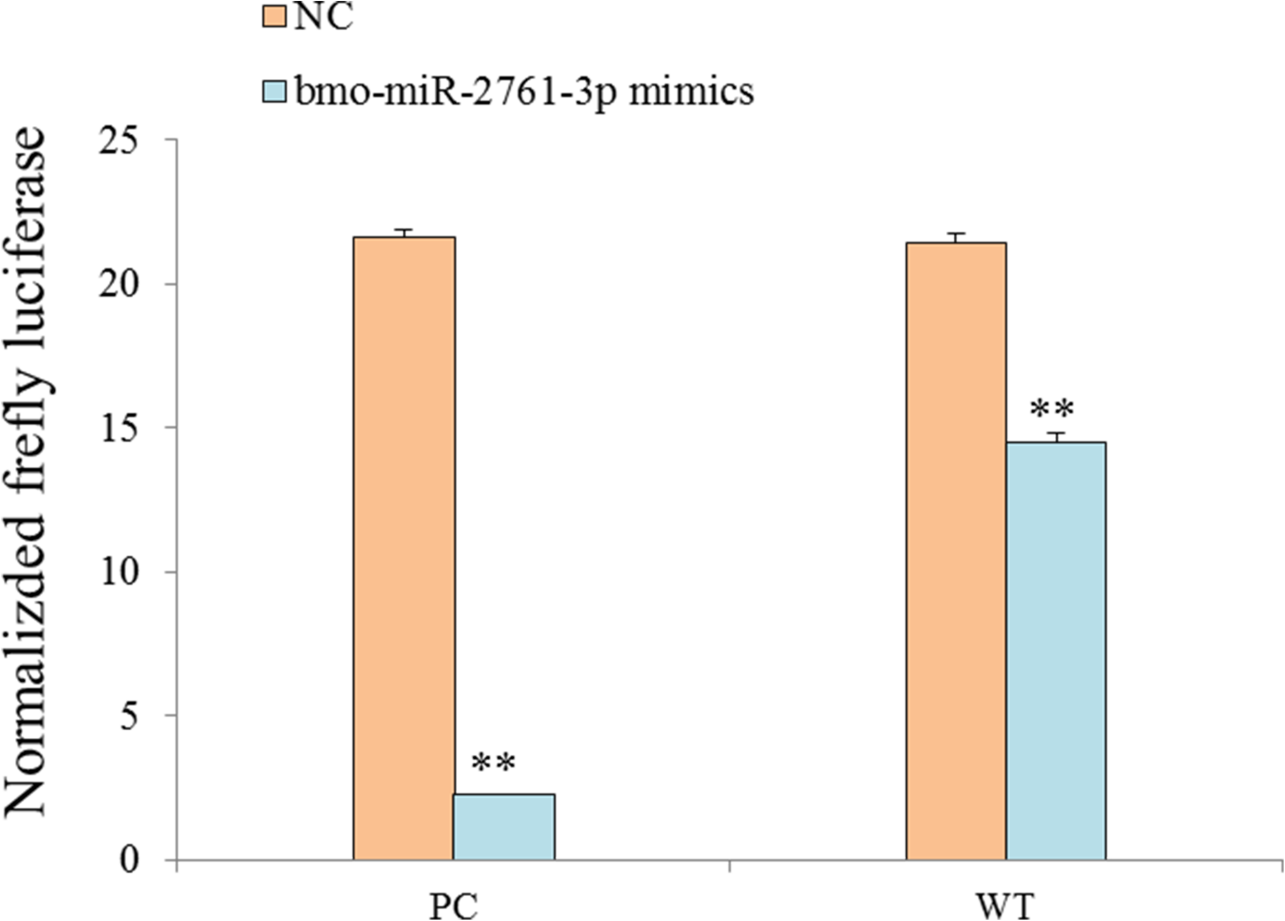
*Bmo-miR-2761-3p* down-regulated *BmSDH* in vitro. PC group represents cells transfected with positive control plasmid (*Bmo-miR-2761-3p* inhibitor inserted into *Luc2* 3’UTR), WT group represents cells transfected with plasmid containing *BmSDH* 3’UTR. “NC” represents cells transfected with the negative control mimics, “mimics” represents cells transfected with *Bmo-miR-2761-3p* mimics. Normalized firefly luciferase activity equals to firefly luciferase activity/Renilla luciferase activity. ** P < 0.01 indicates significant differences compared to the relevant control. Each group was replicated three times using independently collected samples. Error bar = 1 SD.

Target prediction (S2 Fig) and the qRT-PCR result were indicated that *BmSDH* might be one of the target genes of *Bmo-miR-2761-3p*. Base on DLR assay, *BmSDH* 3’UTR was cloned into MCS of pGLo3-constructed WT plasmid and its effect on *Bmo-miR-2761-3p* was measured in HEK293T cells. After a 24-hour co-transfection, the firefly luciferase activity of *Bmo-miR-2761-3p* mimics-treated cells was weaker, approximately 89.6% lower than NC mimics-treated cells in the PC group; while in the WT group, *Bmo-miR-2761-3p* mimic binding *BmSDH* 3’UTR lowered the firefly luciferase expression by 32.4% (Fig 7). Student t-test analysis showed that the expression difference was statistically significant (p<0.01), indicating that *Bmo-miR-2761-3p* could down-regulate the expression of *BmSDH* in vitro.

## Discussion

The molecular mechanism of silkworm diapause remains unclear. In this paper we detected the differentially expressed sRNA between HCl-treated eggs and diapause-destined eggs of 932 strain silkworm. We found that miRNA species increased by 5.15% in response to HCl treatment. 61 differentially expressed and 44 novel miRNAs were identified. Most of the differentially expressed miRNAs increased by 1–3 folds and a few above 5 folds. In general, miRNAs inhibit the genes expression, however, there were 38 miRNAs down-regulated and 23 miRNAs up-regulated post HCl-treated, down-regulated miRNAs were 15 more than up-regulated miRNAs indicated that lower the miRNAs expression would promote genes expression to prevent silkworm egg enter to diapause. Our data suggested that miRNAs may play an important role in the prevention of diapause process.

There are significant differences between diapause and developing embryos. The oxygen consumption falls sharply and the metabolism is seriously suppressed when entering diapause. Enzymes play an important role in the regulation of physiological metabolism. For instance, SDH[4] could prevent the accumulation of sorbitol, which is only presented in the developing embryo of silkworm. And NAD/NADH, NADP/NADPH [7] could regulate respiratory chain. In the present study, we used qRT-PCR to detect the expression of *Bmo-miR-2761-3p* and *BmSDH*, result show that *Bmo-miR-2761-3p* was significantly down-regulated after HCl acid treated, however *BmSDH* mRNA was significantly up-regulated, suggesting that *BmSDH* mRNA expression was closely related to *Bmo-miR-2761-3p*. For further research, we used DLR assay to confirmed that *Bmo-miR-2761-3p* could down-regulate *BmSDH* expression. These results indicated that *Bmo-miR-2761-3p* may suppressed *BmSDH* expression. *BmSDH* is a molecular marker of silkworm embryonic development, and can only be detected in the developing embryo [30]. We hypothesized that high concentration of *Bmo-miR-2761-3p* might inhibit *BmSDH* expression, maintaining diapause stage. With HCl treatment, *Bmo-miR-2761-3p* was down-regulated and *BmSDH* was activated, which then started glycogen synthesis and mediated changes of metabolism to promote embryogenesis.

To further understand the regulation of miRNAs in *B*.*mori* diapause, the DE miRNAs target genes were predicted. The CT genes were enriched to GO database. GO annotation of the CT genes indicated that DE miRNAs might participated in protein formation, process and transportation, and regulation of catalytic activities of enzymes. These target genes are closely related to protein synthesis and metabolic function. In the previous studies, researcher suggesting the highly expressed *Bmo-miR-2* in the egg stage possibly related to the generation of embryos [8]. Another highly expressed miRNA *Bmo-miR-iab-4-3p* can targets Ser/Thr kinase, which regulates G2/M2 transition, it was believed that play an important role during embryo development [31,32]. In combination with these results we have reason to believe that these DE miRNAs might impact embryo development by metabolic regulation. The CT genes also enriched to KEGG pathway database. KEGG Pathway significant enrichment can determine the candidate target gene involved in the most important biochemical metabolic pathway and signal transduction pathway. Our data from KEGG pathway enrichment suggested that CT genes mainly involved in the protein processing in endoplasmic reticulum pathway and Spliceosome pathway, these two pathways affect mRNA cleavage and protein synthesis, which is essential for the development of silkworm eggs. In addition, the special miRNAs could regulated the metabolism of Gly and Ser/Thr, and might also participate in mTOR, Notch signal pathway that regulates cell cycle, division and differentiation. As such, DE miRNAs might decide silkworm embryo fate through cell cycle regulation. By comparing the differentially expressed miRNAs between the control and HCl-treated eggs, we found that 2 miRNAs appeared after HCl treatment and were not detected in control eggs and 4 miRNAs disappeared after HCl treatment, while 5 miRNAs had much higher expression than other DE miRNAs (Table 2). Therein, *novel_45/novel_48/Bmo-miR-2809/3228/3384-3p* had potential targets in Notch 3’UTR, which is a key regulatory factor of NOTCH pathway. *Bmo-miR-3384-3p* targeted NLK, *novel_45/Bmo-miR-3228/3274/6497-3p* targeted Beta-catenin and *novel_48/bmo-miR-3384-3p/ 6497-3p* targeted Wnt. These target genes NLK/Beta-catenin/Wnt both play an important role in WNT pathway. *Bmo-miR-3384-3p/6497-3p* might regulate MAPK pathway through targeting *Ras1*. And the MAPK pathway was reported that might play an important role in diapause termination. All these specific miRNAs might be the essential regulated factors for *Bombyx* embryonic development. We also detected that *let7* family miRNA *Bmo-let-7-5p* was significantly up-regulated. As it is widely recognized that *let7* can regulate the embryonic development, *Bmo-let-7-5p* may be a regulator in the termination of diapause process.

## Conclusions

Summarily, this study provides the sRNA length distribution, components and the expression pattern of miRNAs between diapause-destined and HCl acid treated silkworm eggs. Preliminary compared the expression levels of miRNAs between HCl-treated and diapause eggs. The GO and KEGG pathway enrich analysis of the DE miRNAs target genes provided us the potential molecular mechanisms of miRNA in the silkworm embryo. qRT-PCR and the DLR assay results indicated *Bmo-miR-2761-3p* can downregulated the *BmSDH* expression. These results will also be essential to further research on molecular basis of diapause in Lepidoptera insects. However, this study was initial, the more different pattern of miRNAs in each silkworm embryo stage and the function of DE miRNAs function should be in-depth analysis.

## Supporting information

S1 Fig Dual luciferase expression vector pmirGlo. (TIF)

S2 Fig The predicted of *Bmo-miR-2761-3p* target in *BmSDH*. (TIF)

S1 Table sRNA distribution. (XLS)

S2 Table The sequence of novel miRNAs. (XLS)

S3 Table Up-regulated miRNAs. (XLS)

S4 Table Down-regulated miRNAs. (XLS)

S1 File Secondary structure for novel miRNAs. (DOC)
