# Supplementary figures and images for: Differentially expressed microRNAs in diapausing versus HCl-treated Bombyx embryos
Source: PLoS One. 2017 Jul 11;12(7):e0180085. doi: 10.1371/journal.pone.0180085 (PMC5507411; doi:10.1371/journal.pone.0180085)

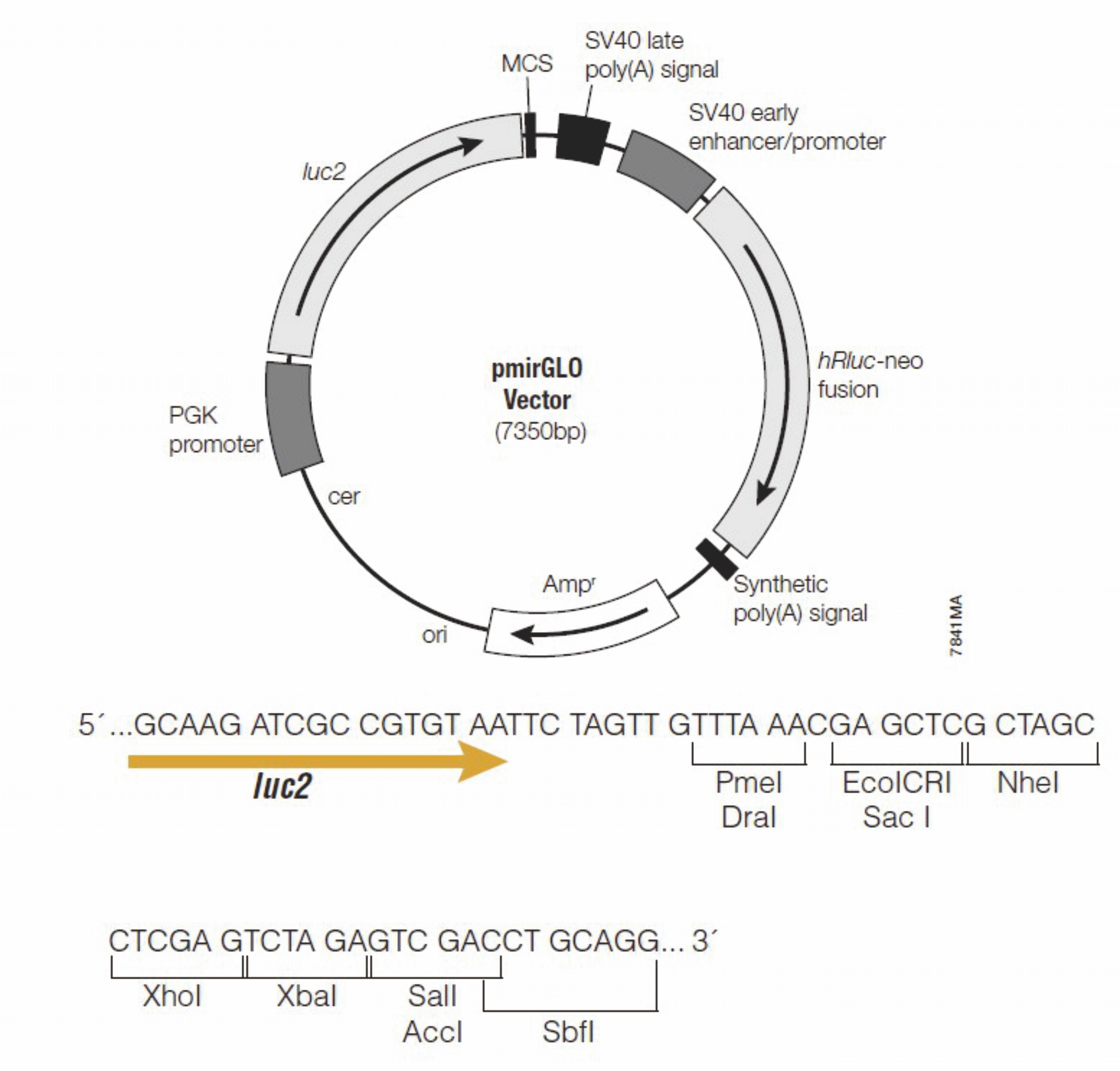

Supplement: S1 Fig — (TIF) [file pone.0180085.s001.tif]

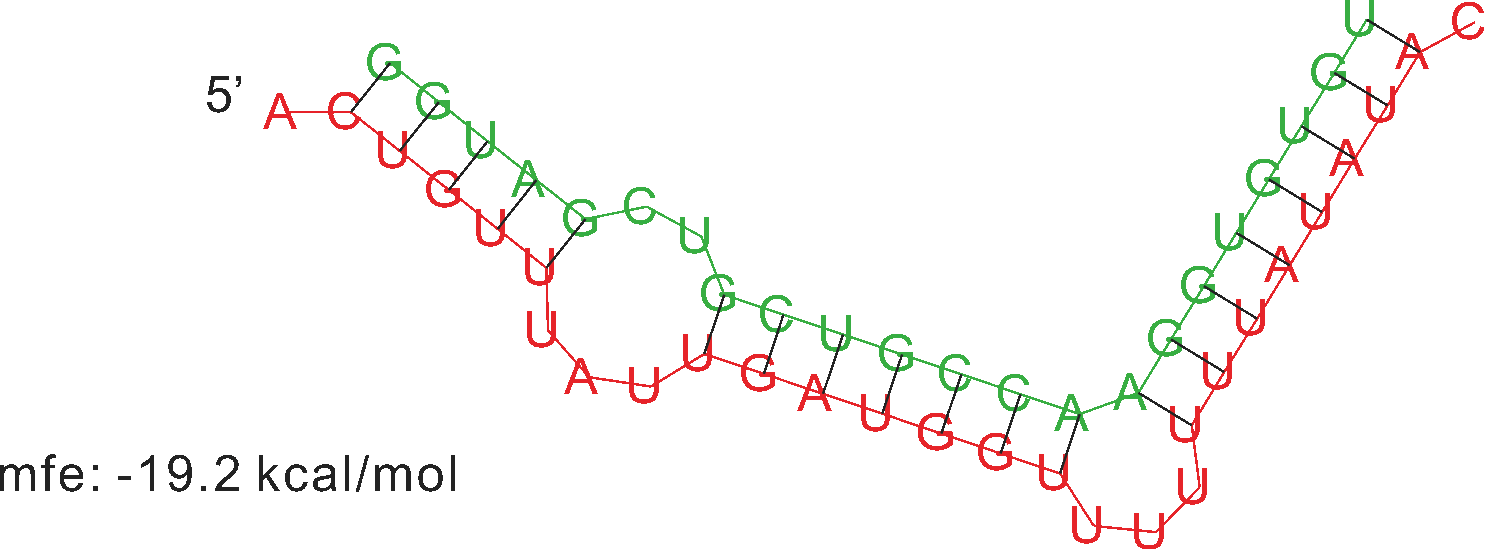

Supplement: S2 Fig — (TIF) [file pone.0180085.s002.tif]
