# Supplementary material for: Differentially expressed microRNAs in diapausing versus HCl-treated Bombyx embryos
Source: PLoS One. 2017 Jul 11;12(7):e0180085. doi: 10.1371/journal.pone.0180085 (PMC5507411; doi:10.1371/journal.pone.0180085)

Supporting Information


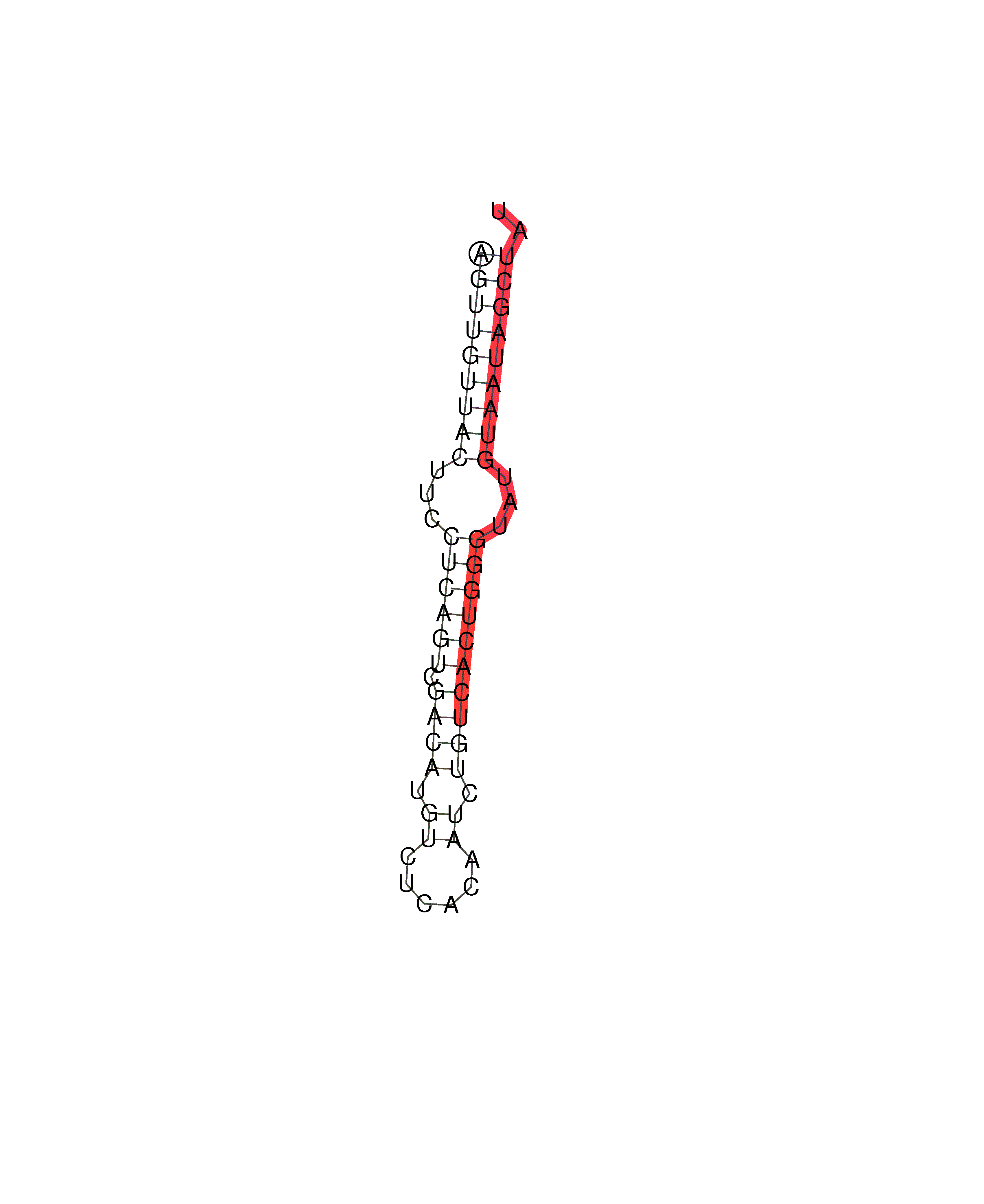
A Fig. Secondary structure for novel_1


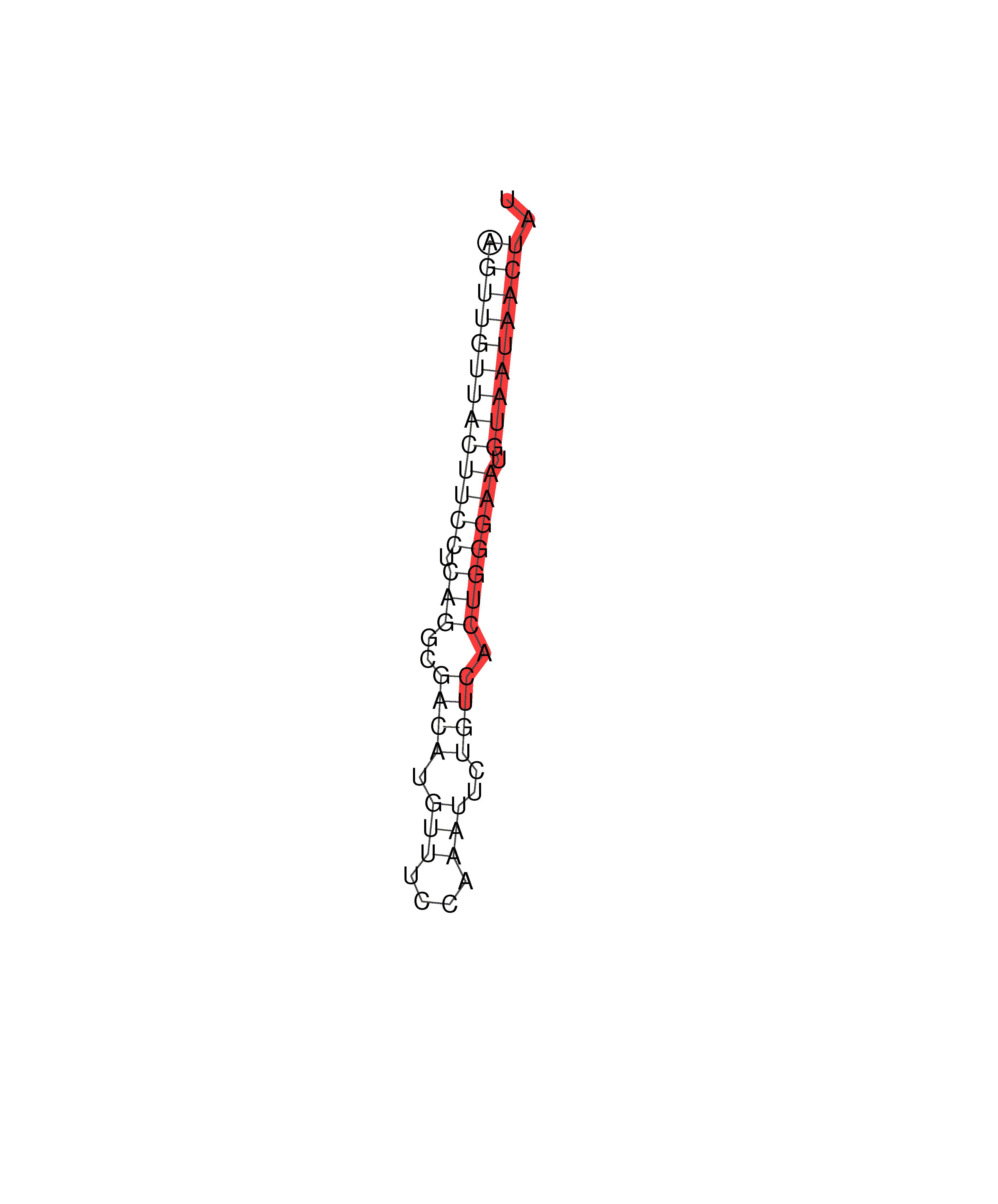


B Fig. Secondary structure for novel_2


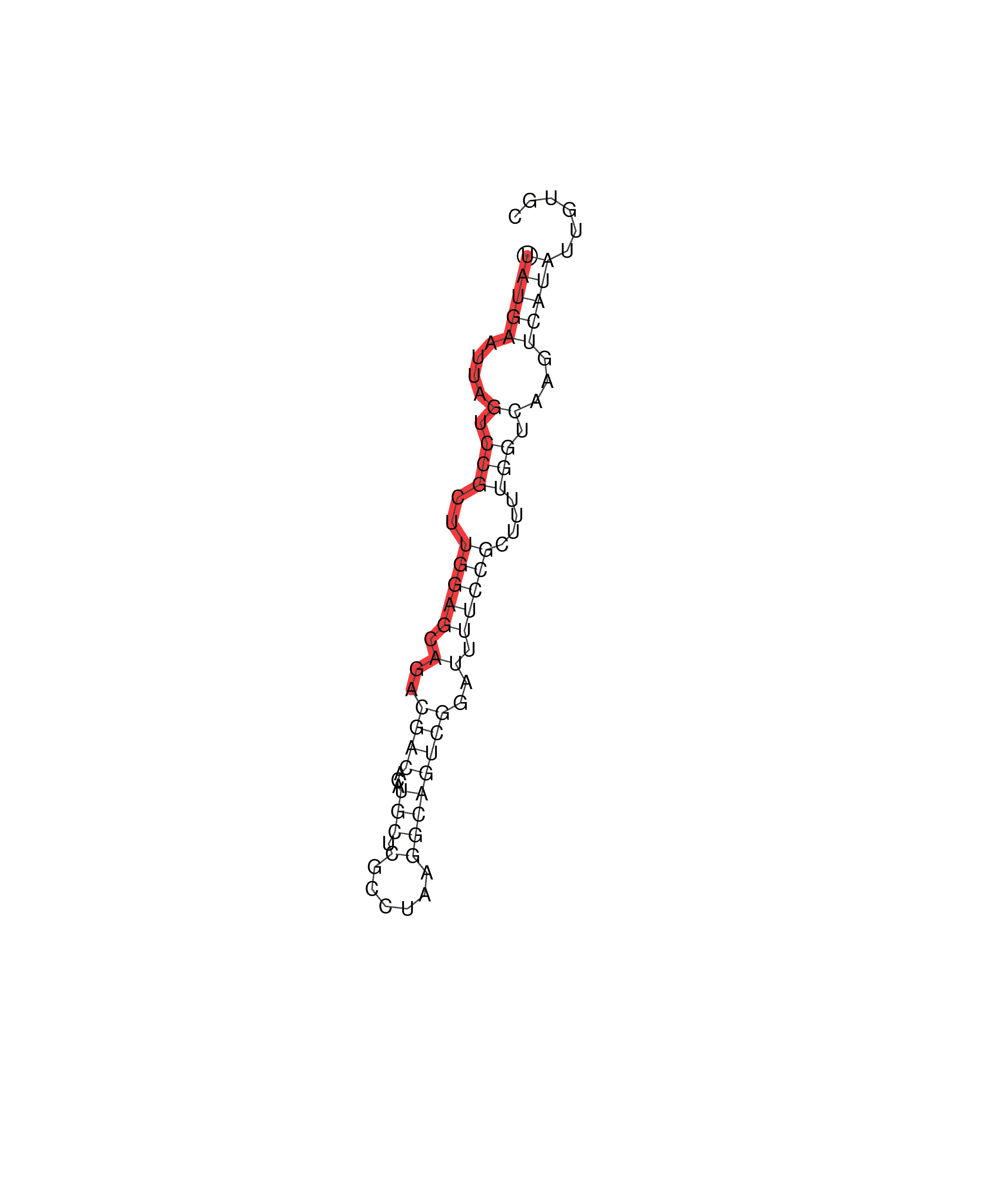


C Fig. Secondary structure for novel_3


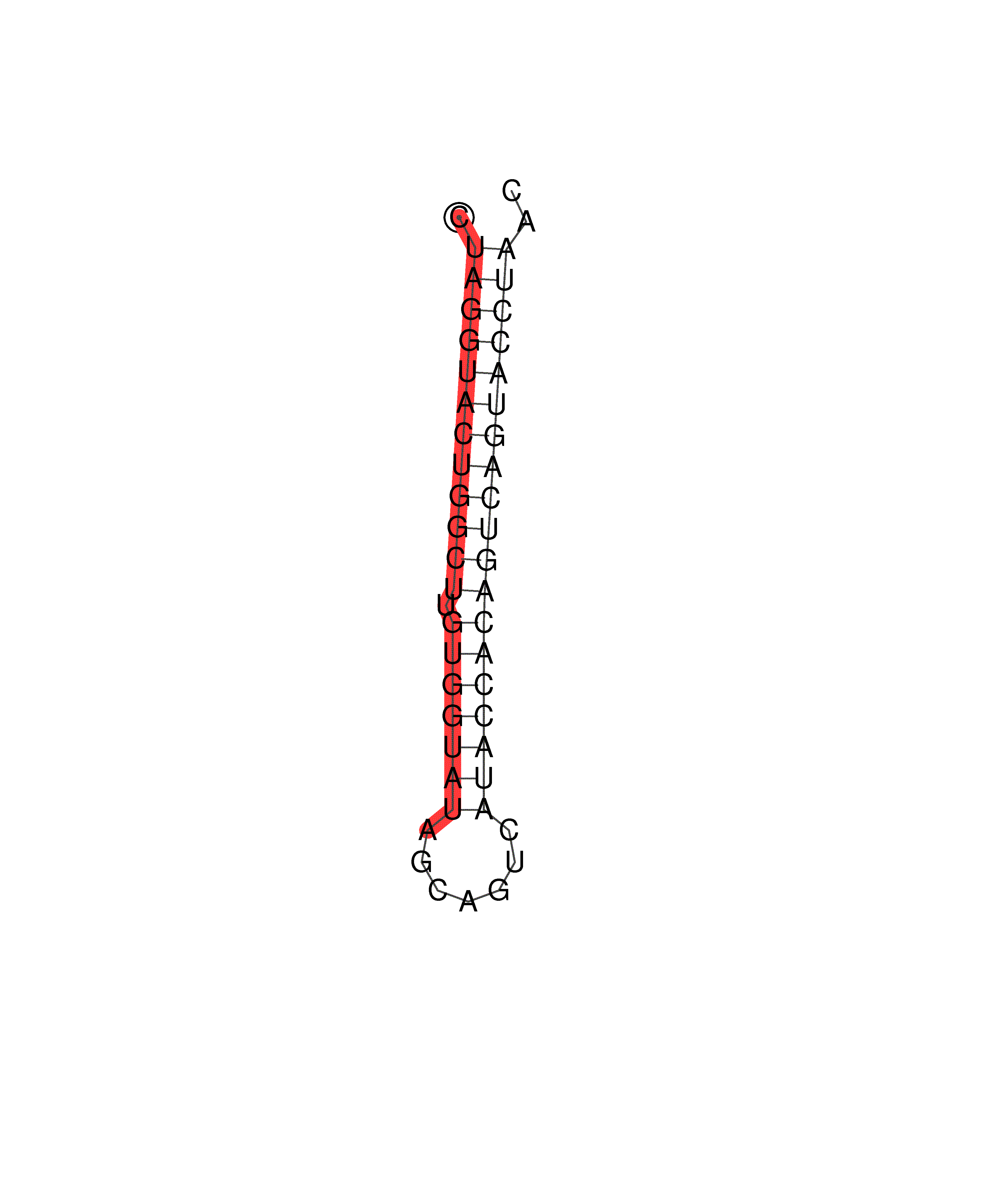
D Fig. Secondary structure for novel_4

E Fig. Secondary structure for novel_8


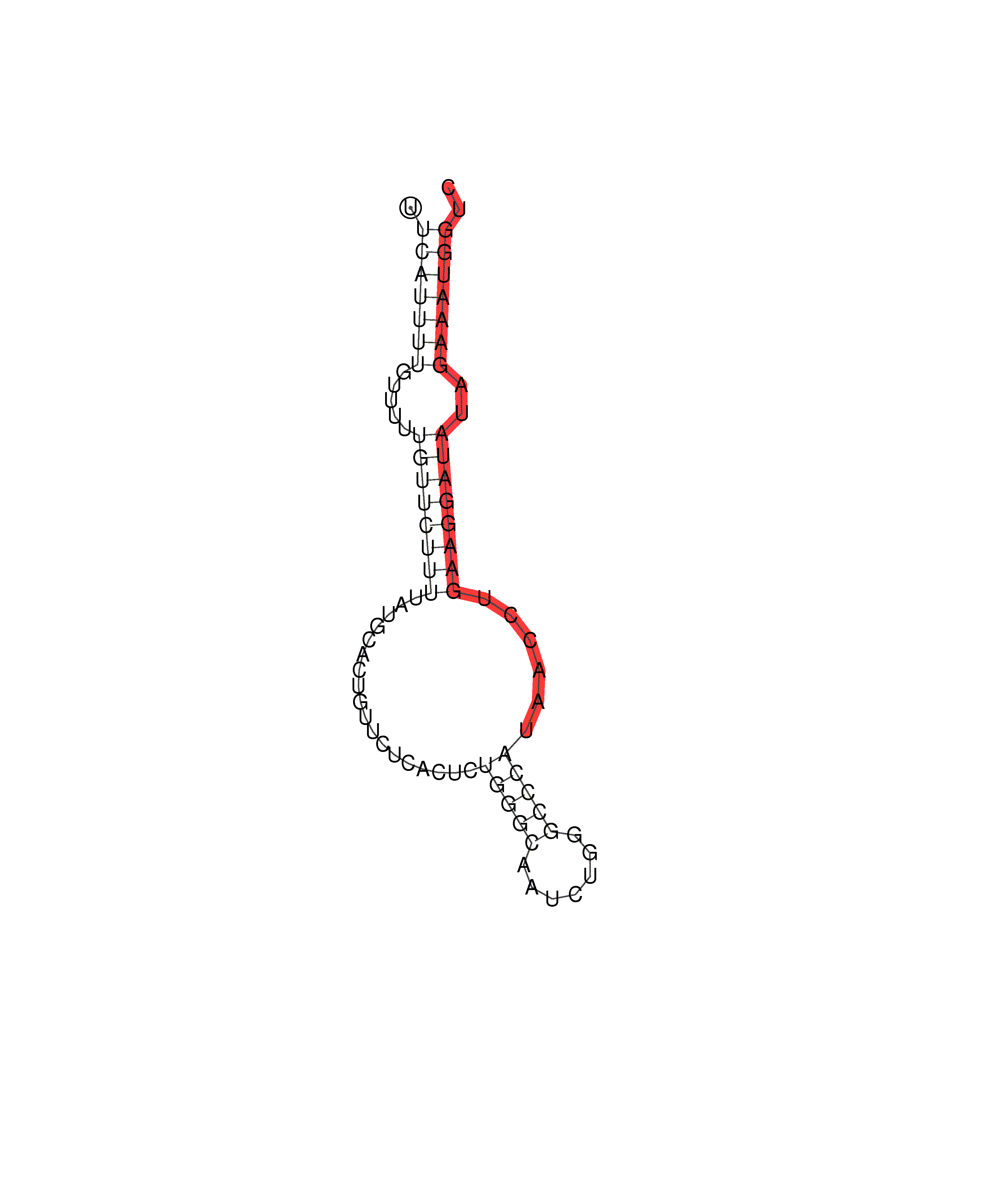


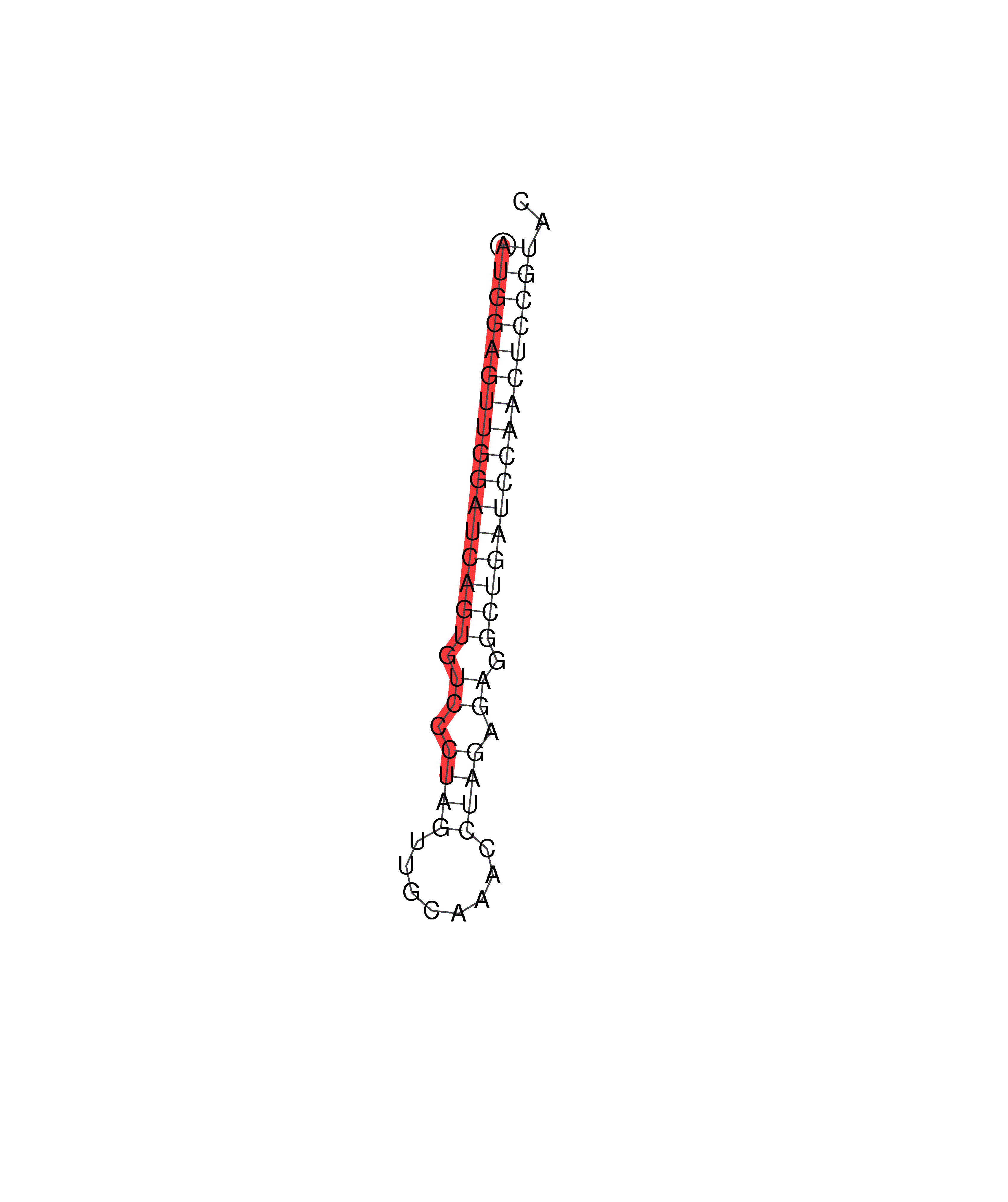
F Fig. Secondary structure for novel_9

G Fig. Secondary structure for novel_10


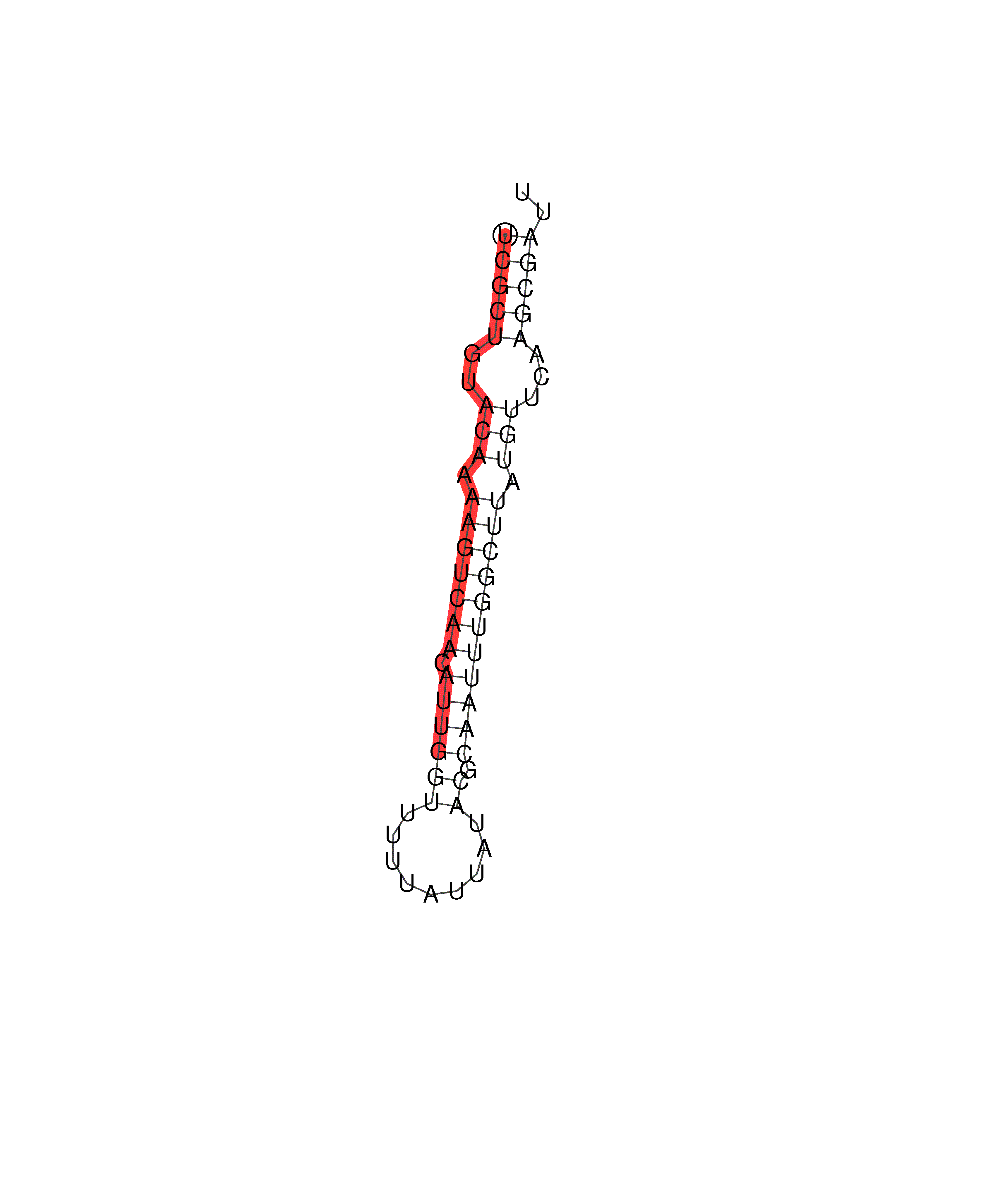


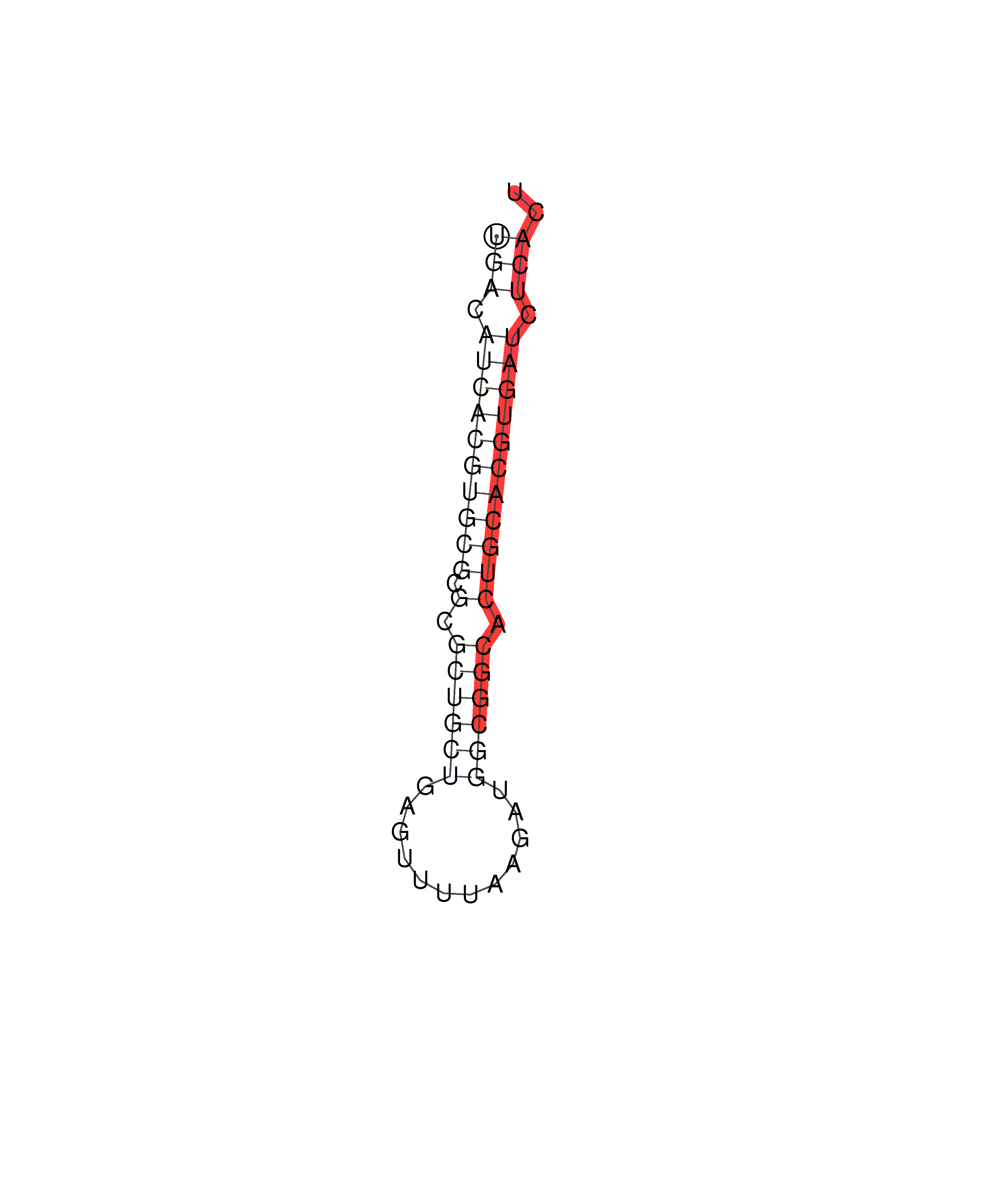
H Fig. Secondary structure for novel_11


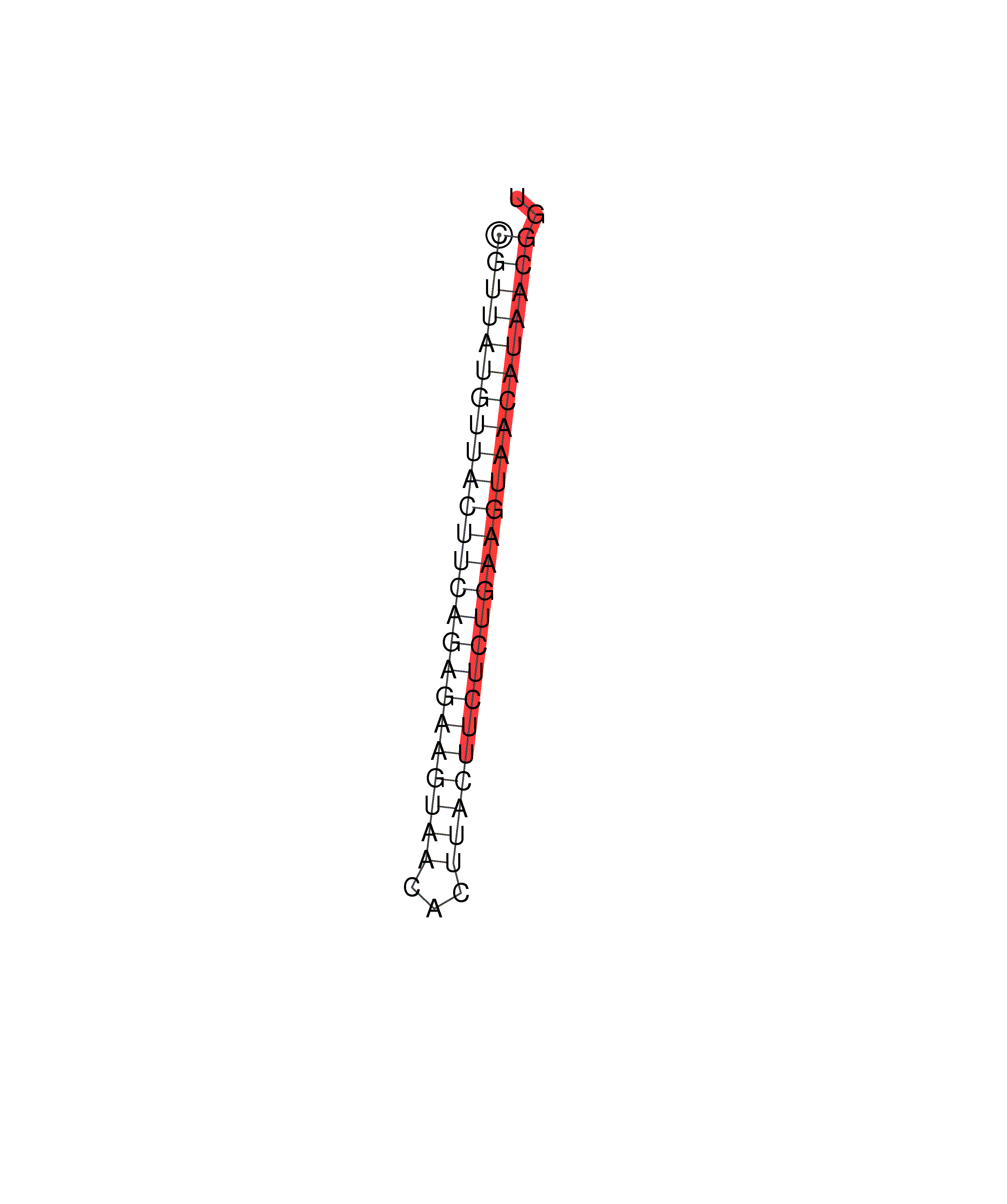


I Fig. Secondary structure for novel_12

J Fig.
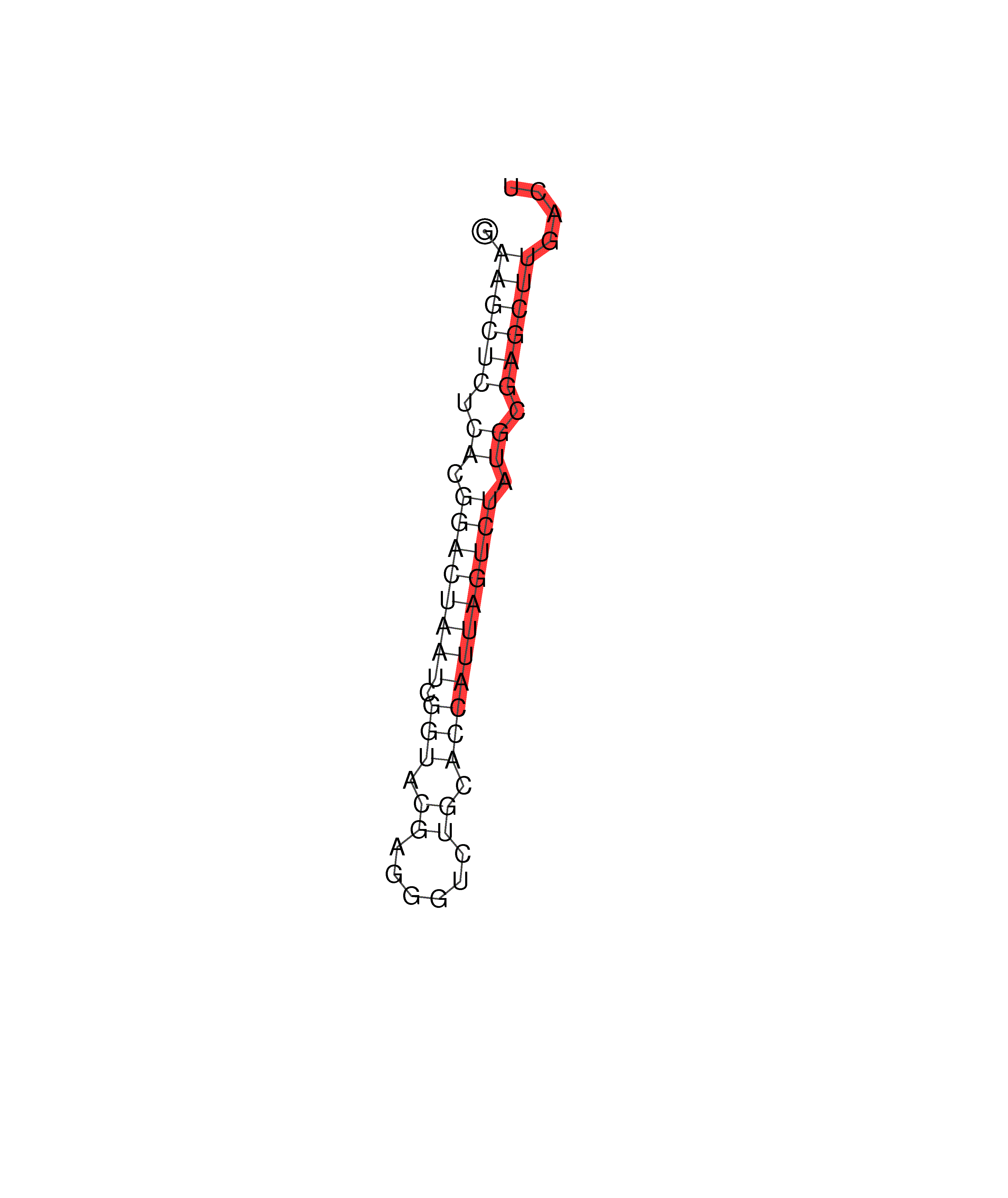
Secondary structure for novel_14


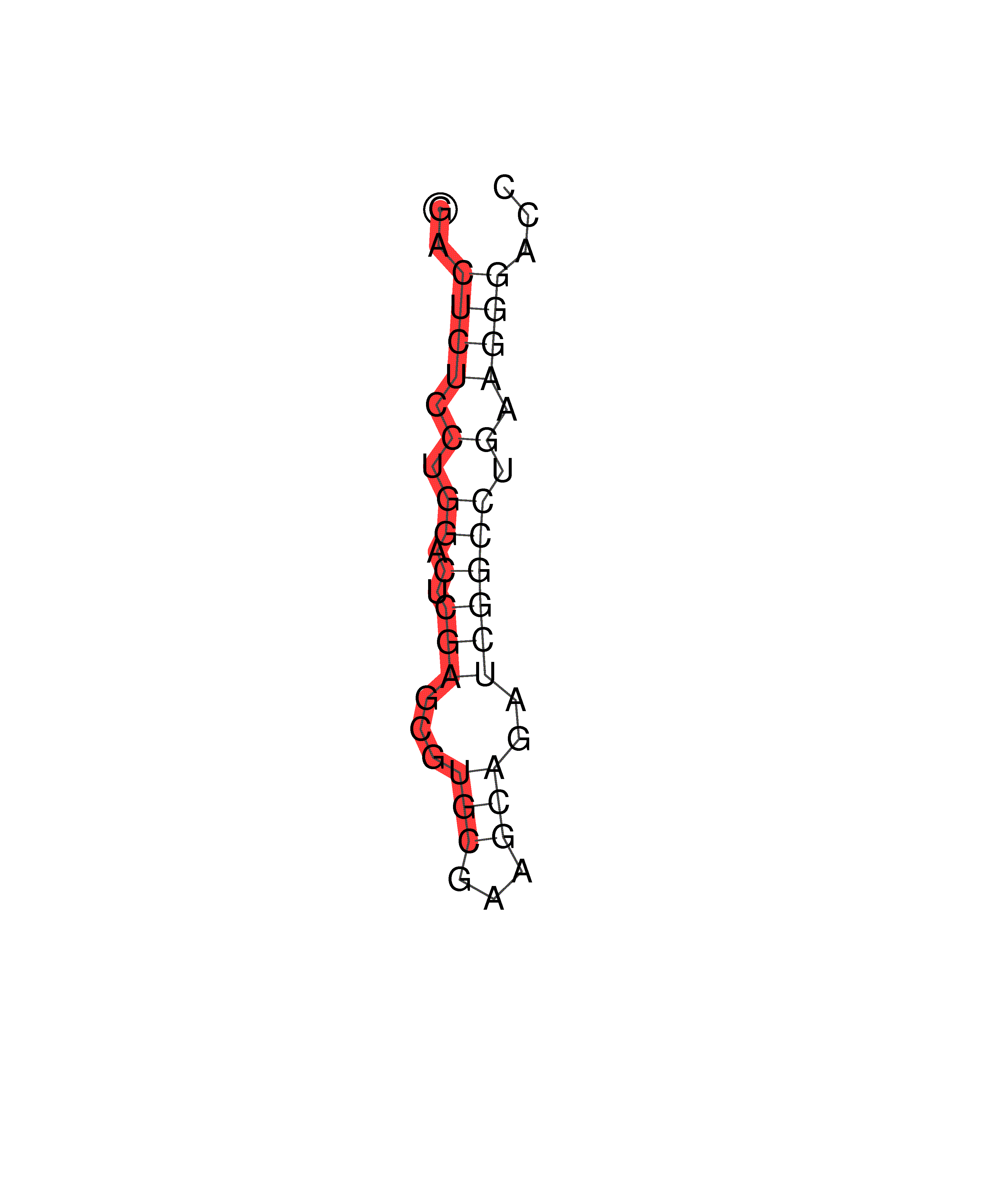


K Fig. Secondary structure for novel_15

L Fig.
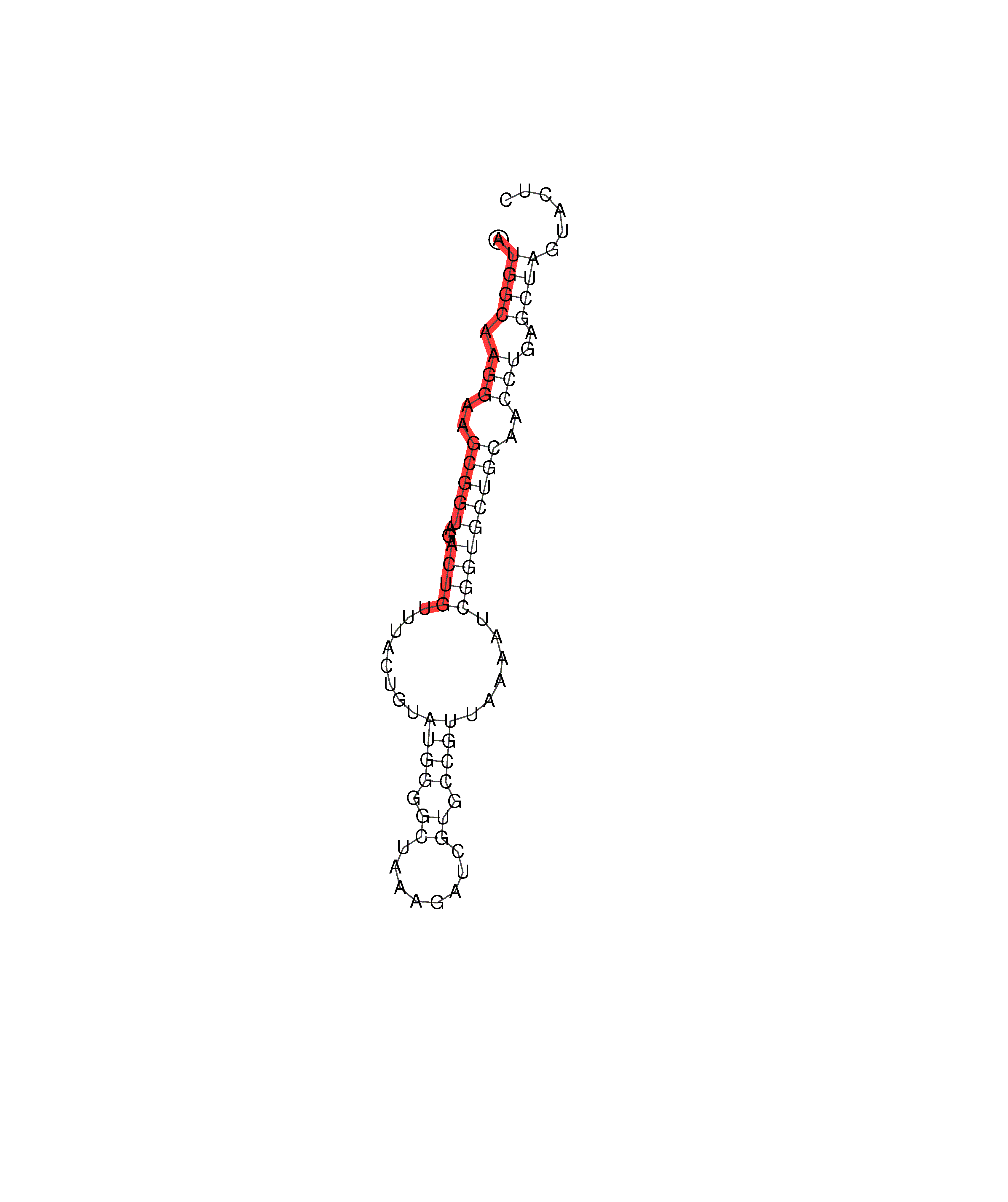
Secondary structure for novel_16


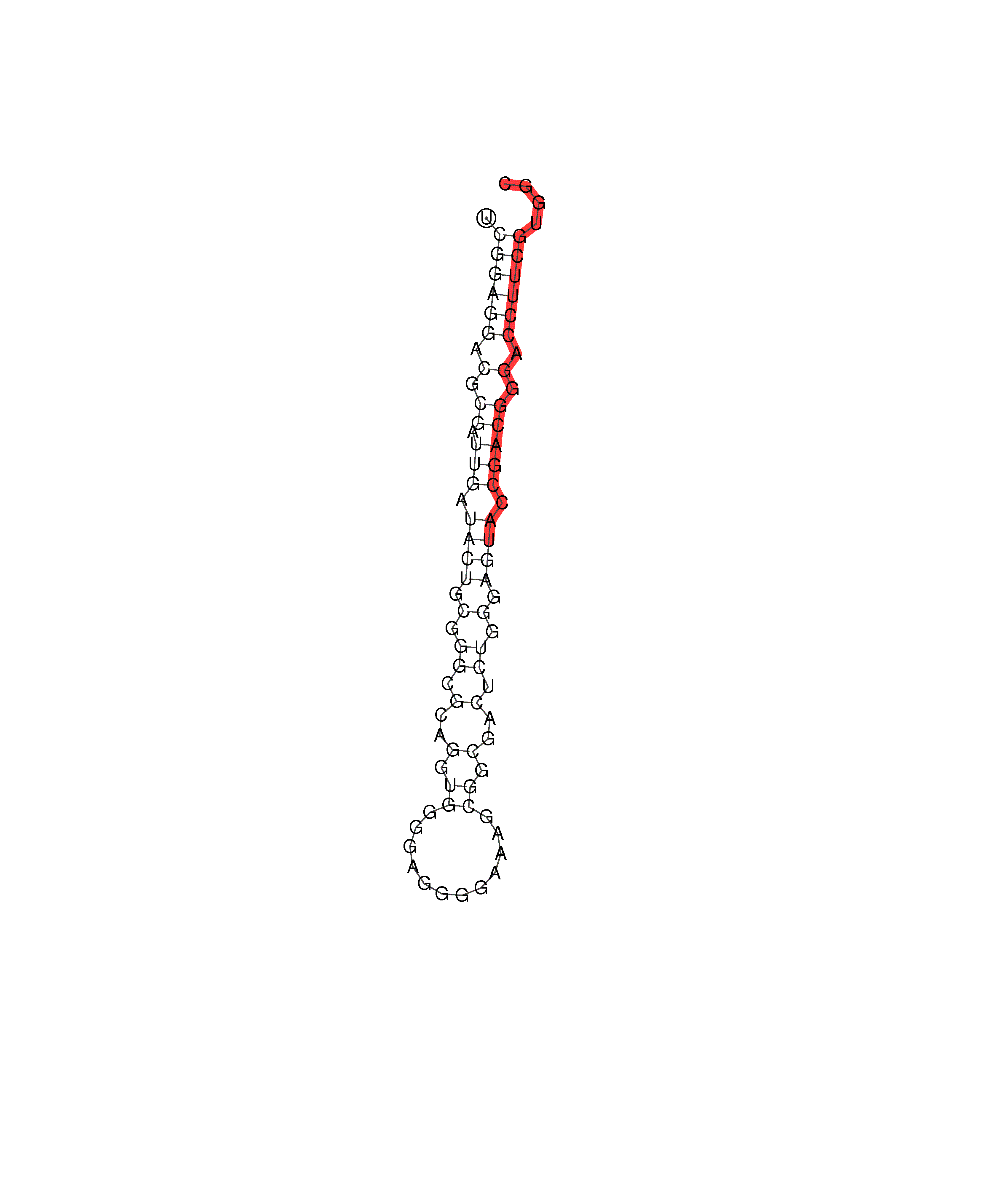


M Fig. Secondary structure for novel_19

N Fig.
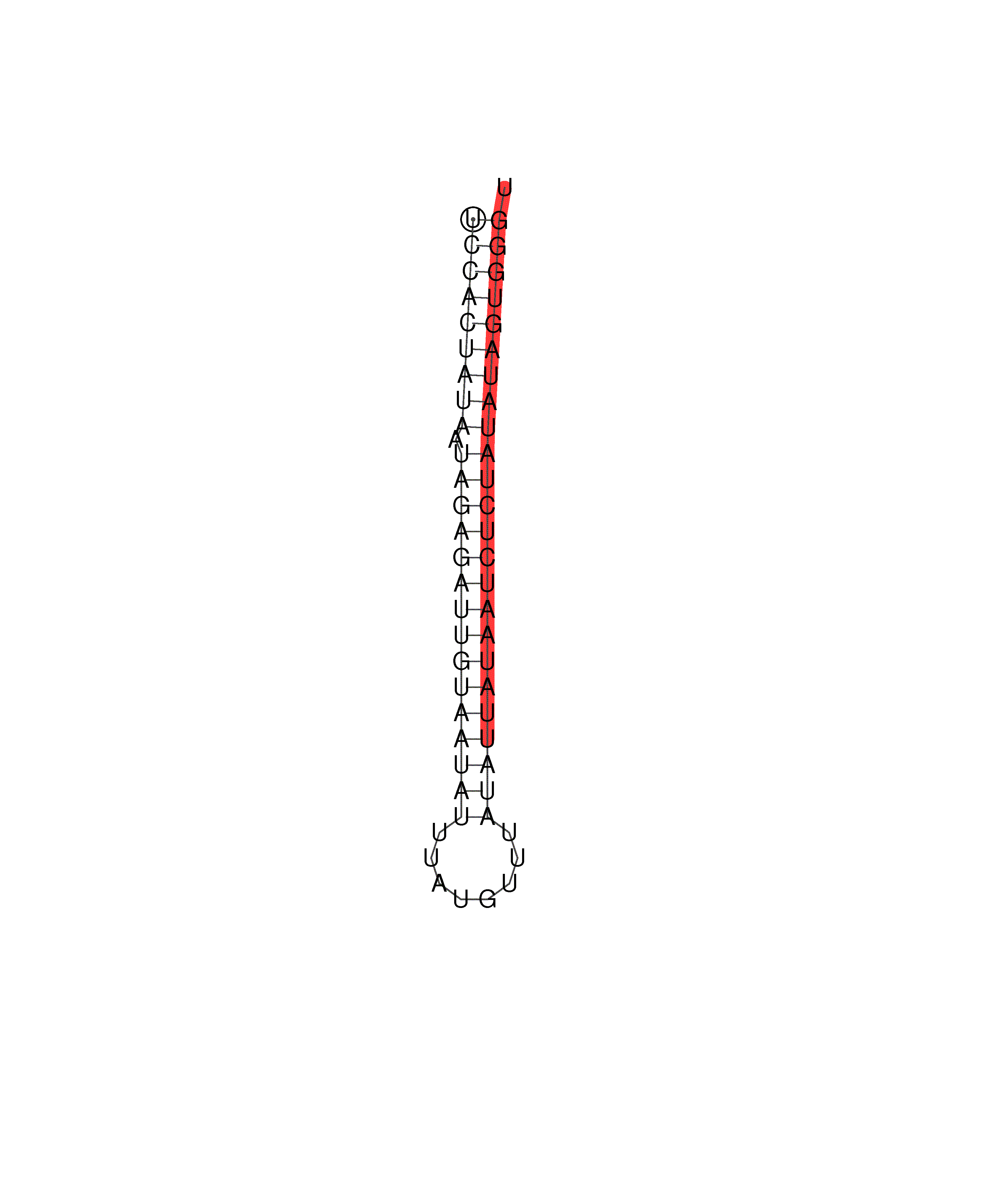
Secondary structure for novel_20


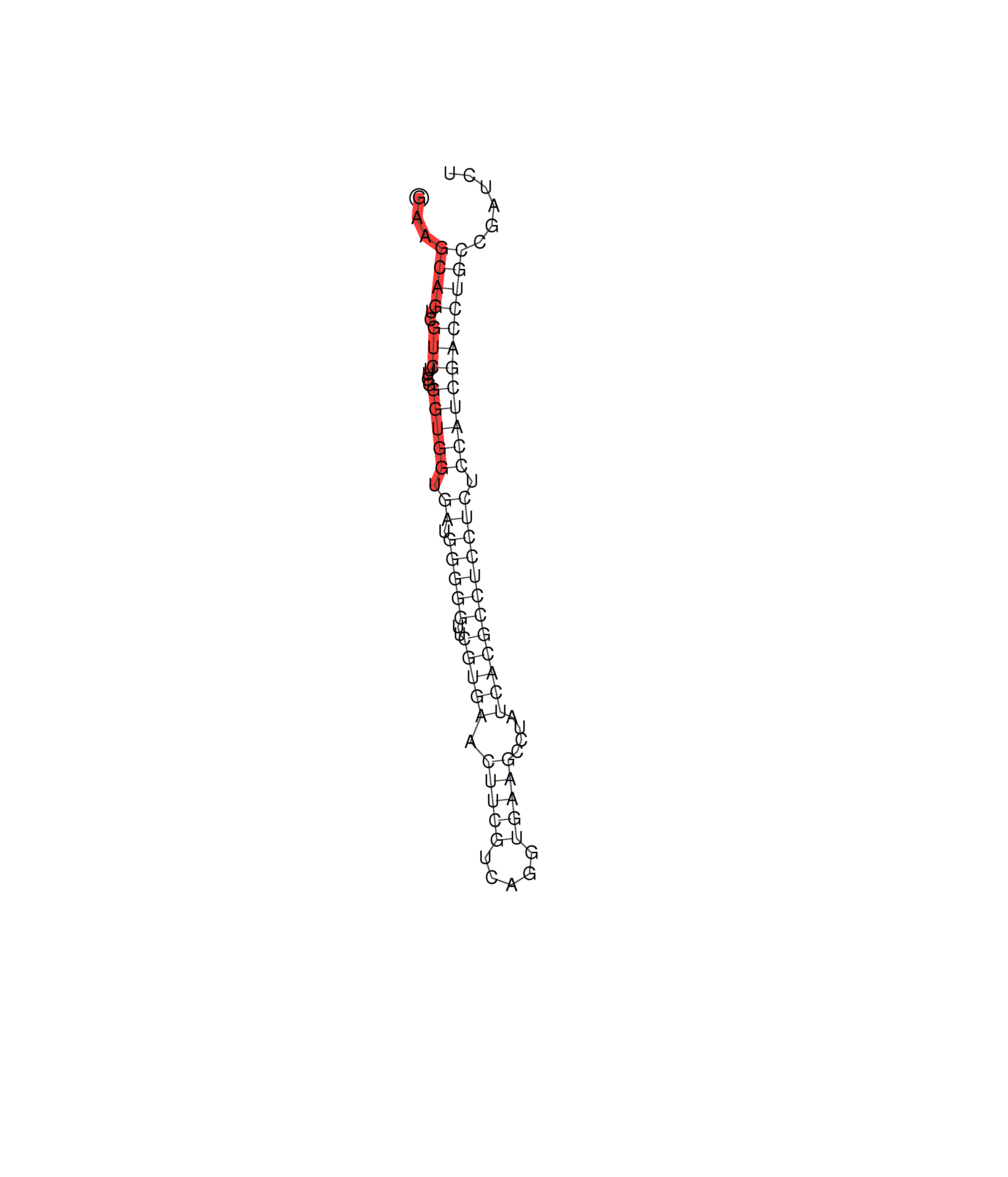


O Fig. Secondary structure for novel_21

P Fig.
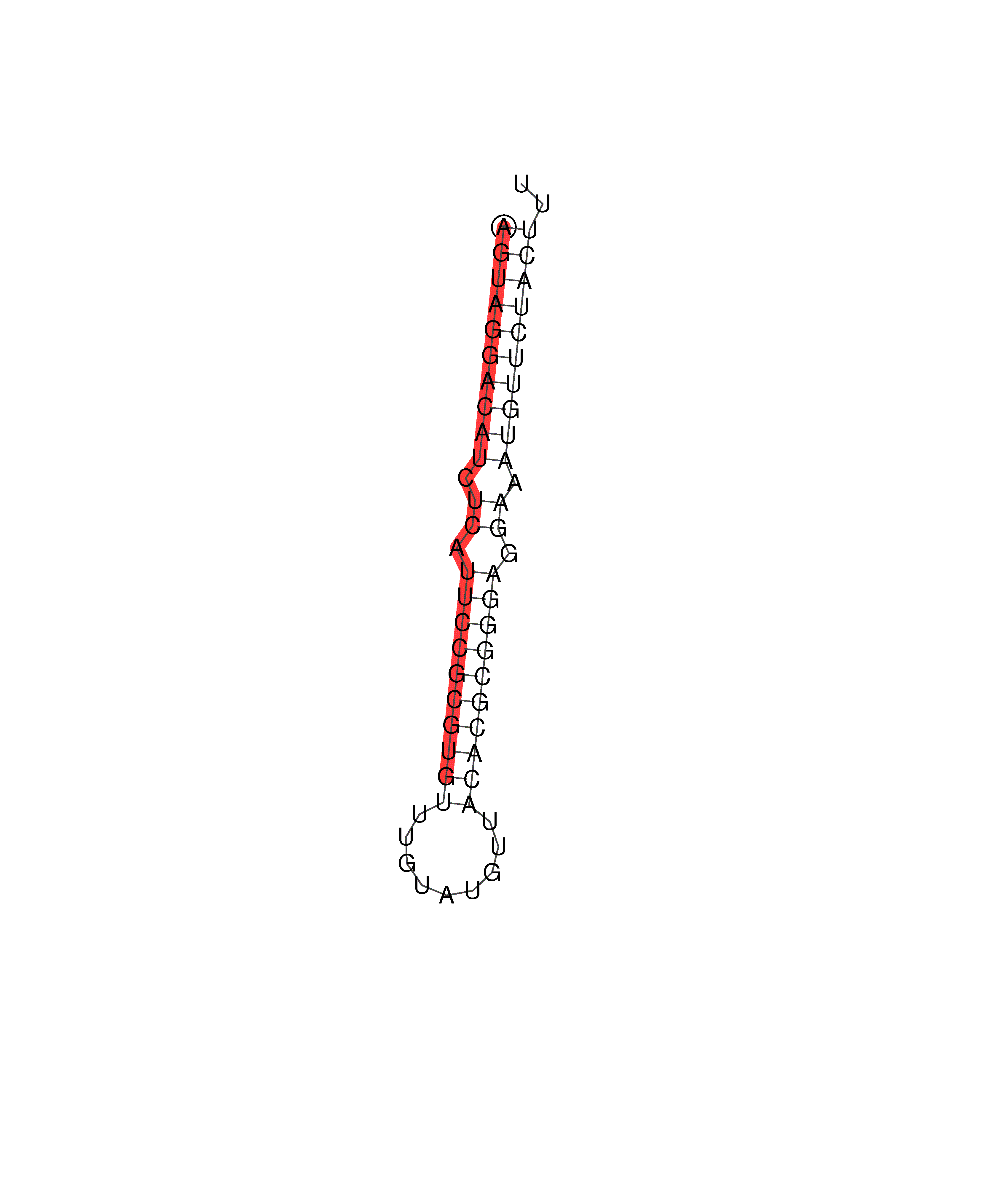
Secondary structure for novel_22

Q Fig.
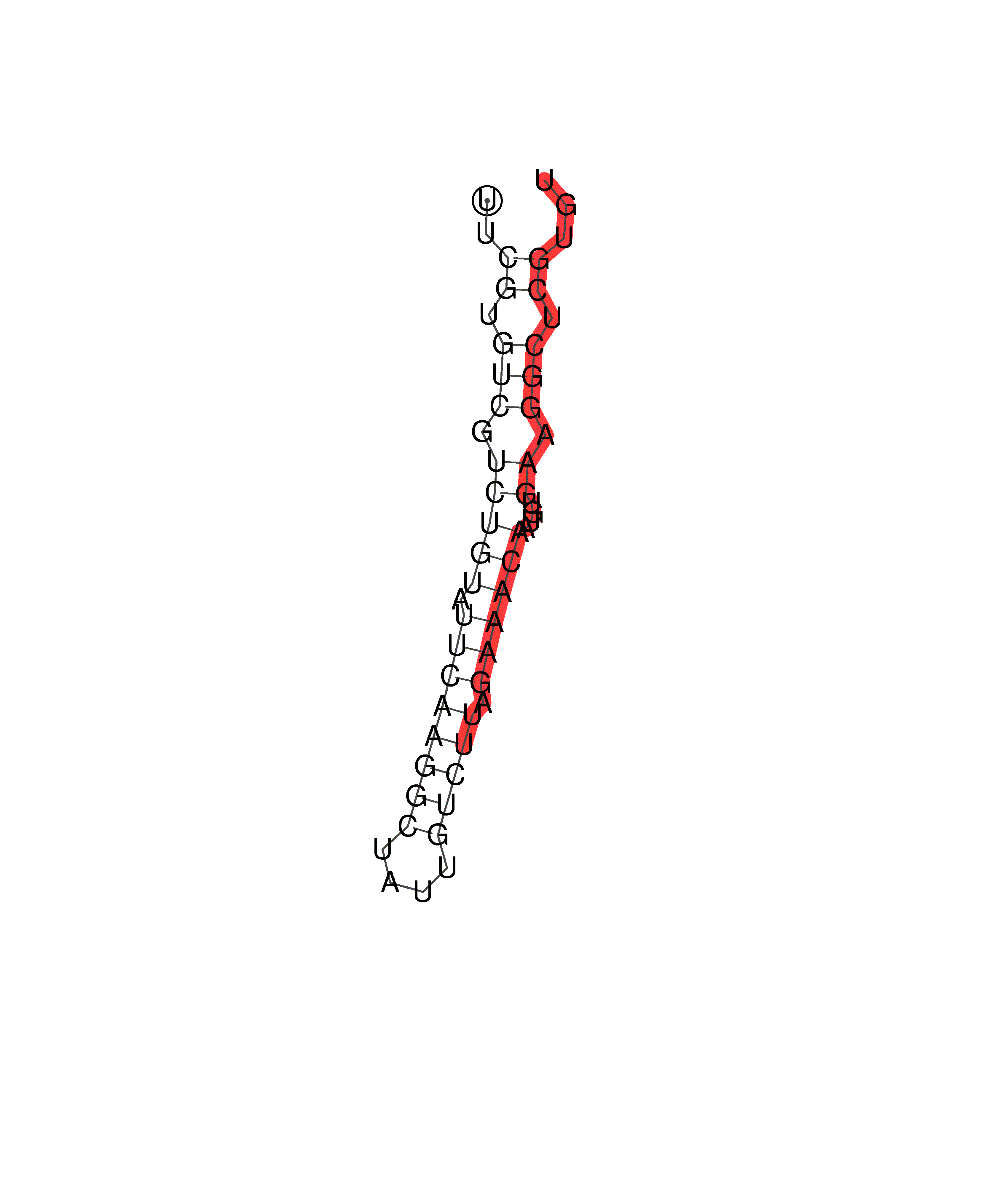
Secondary structure for novel_23

R Fig.
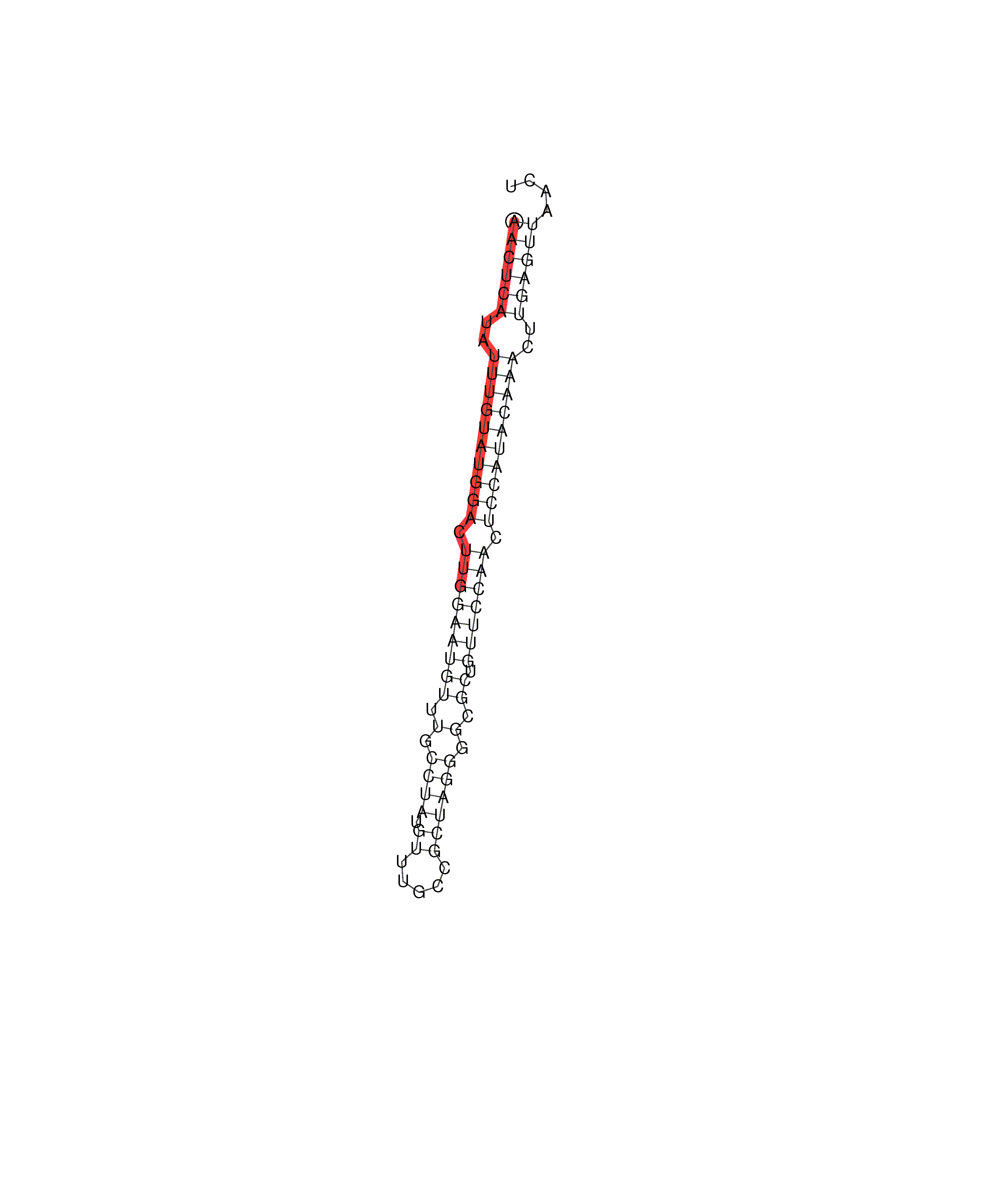
Secondary structure for novel_24


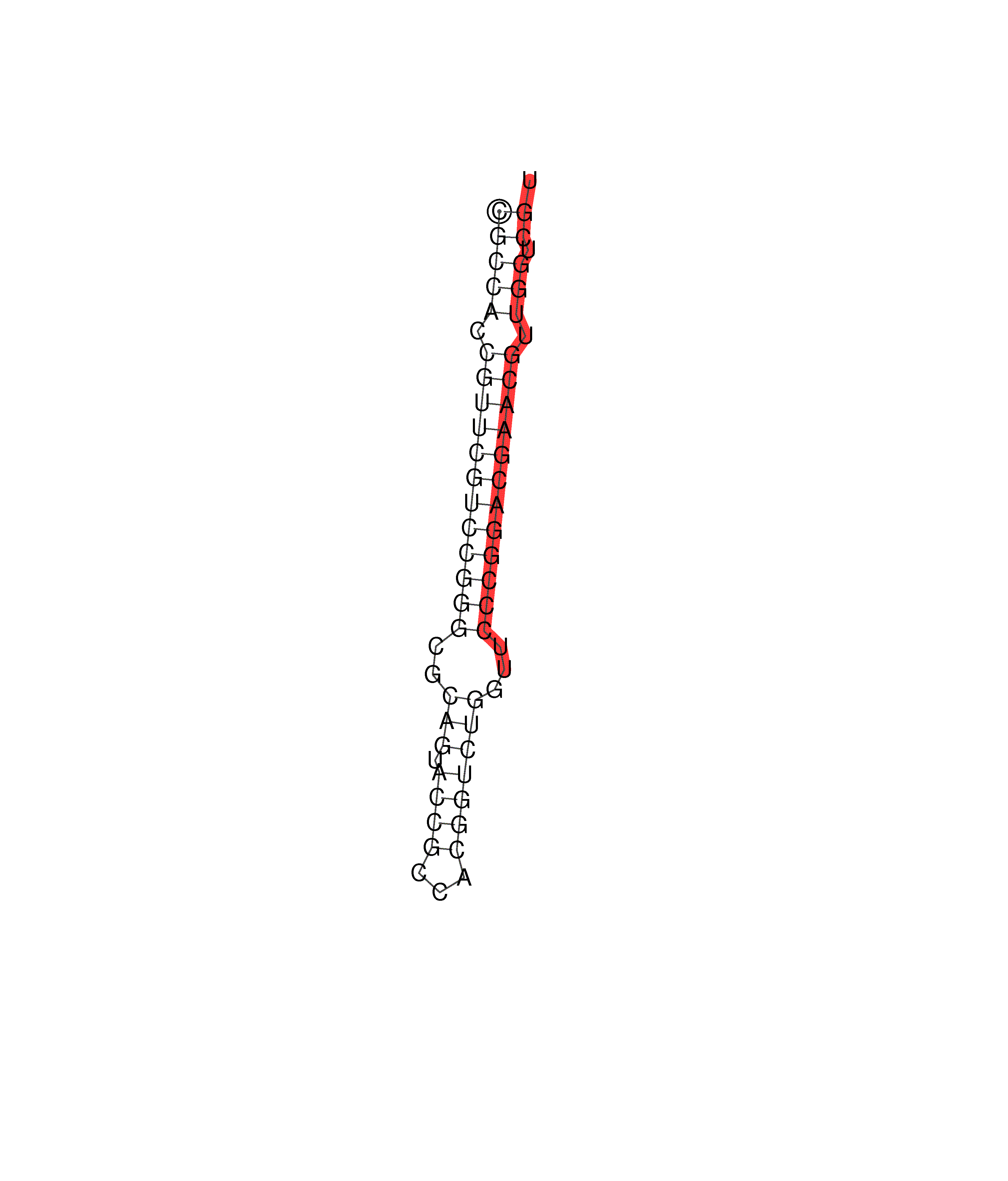


S Fig. Secondary structure for novel_25

Y Fig.
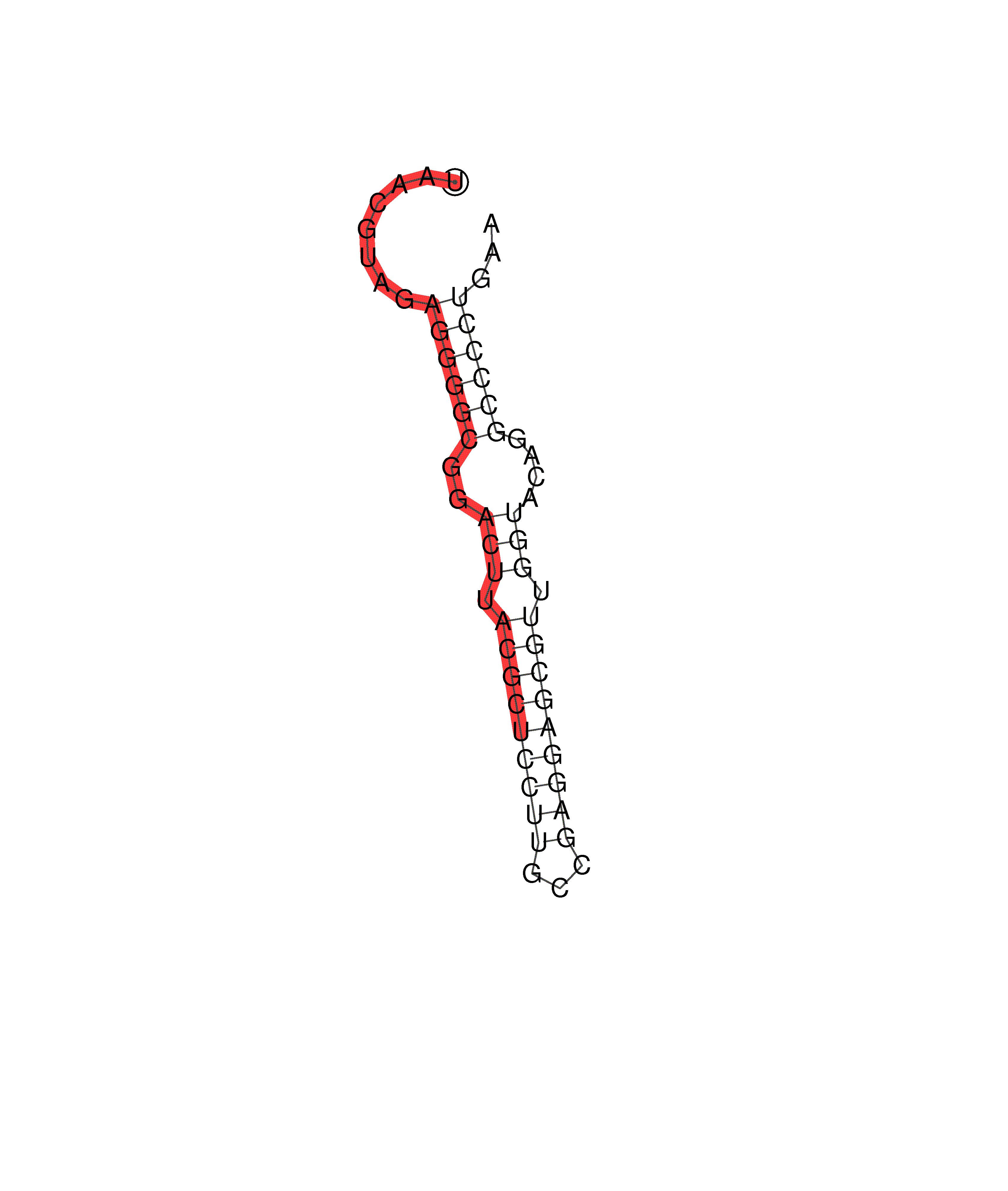
Secondary structure for novel_26


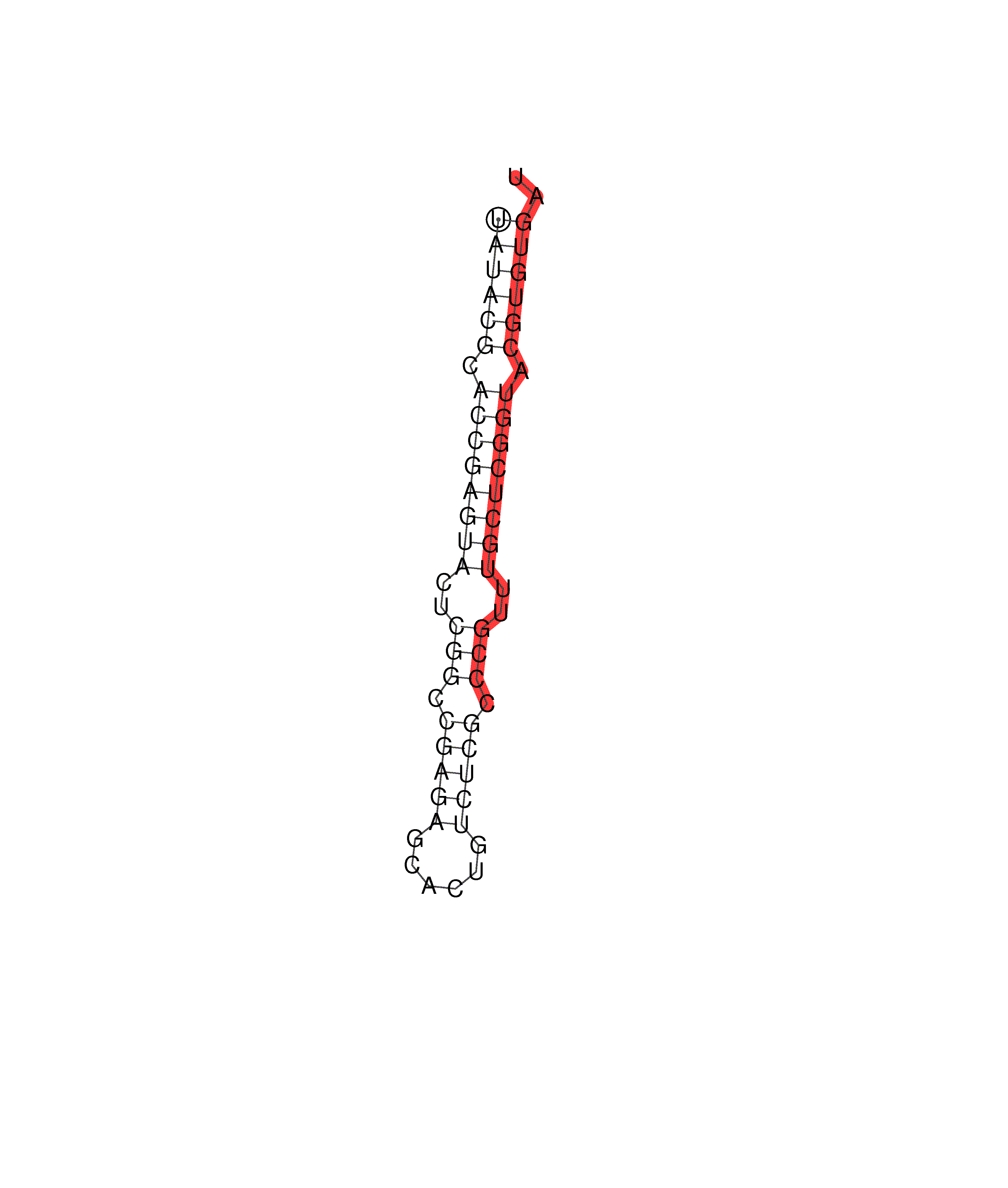


U Fig. Secondary structure for novel_29

V Fig.

Secondary structure for novel_30

W Fig.

Secondary structure for novel_31

X Fig.
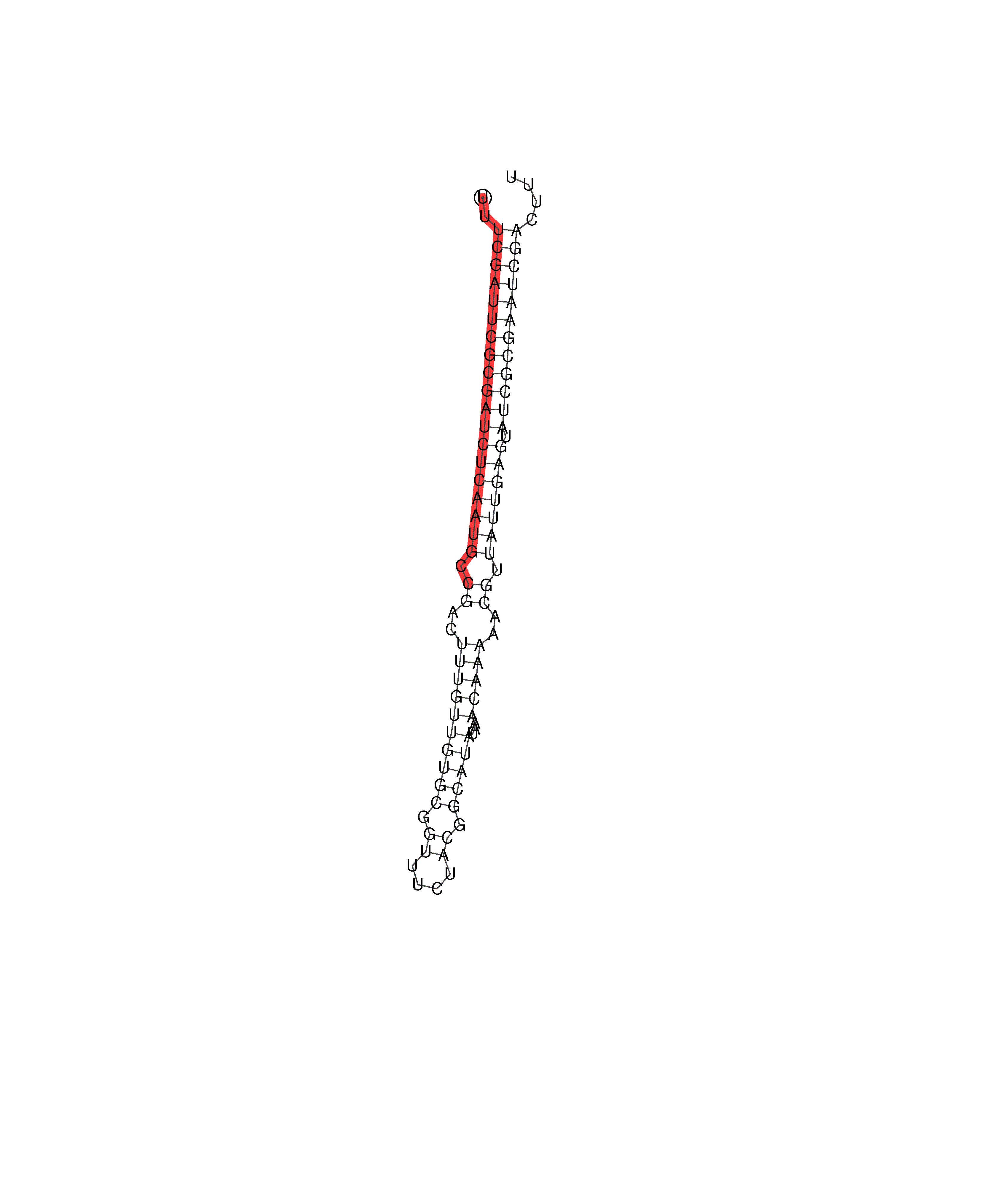
Secondary structure for novel_32


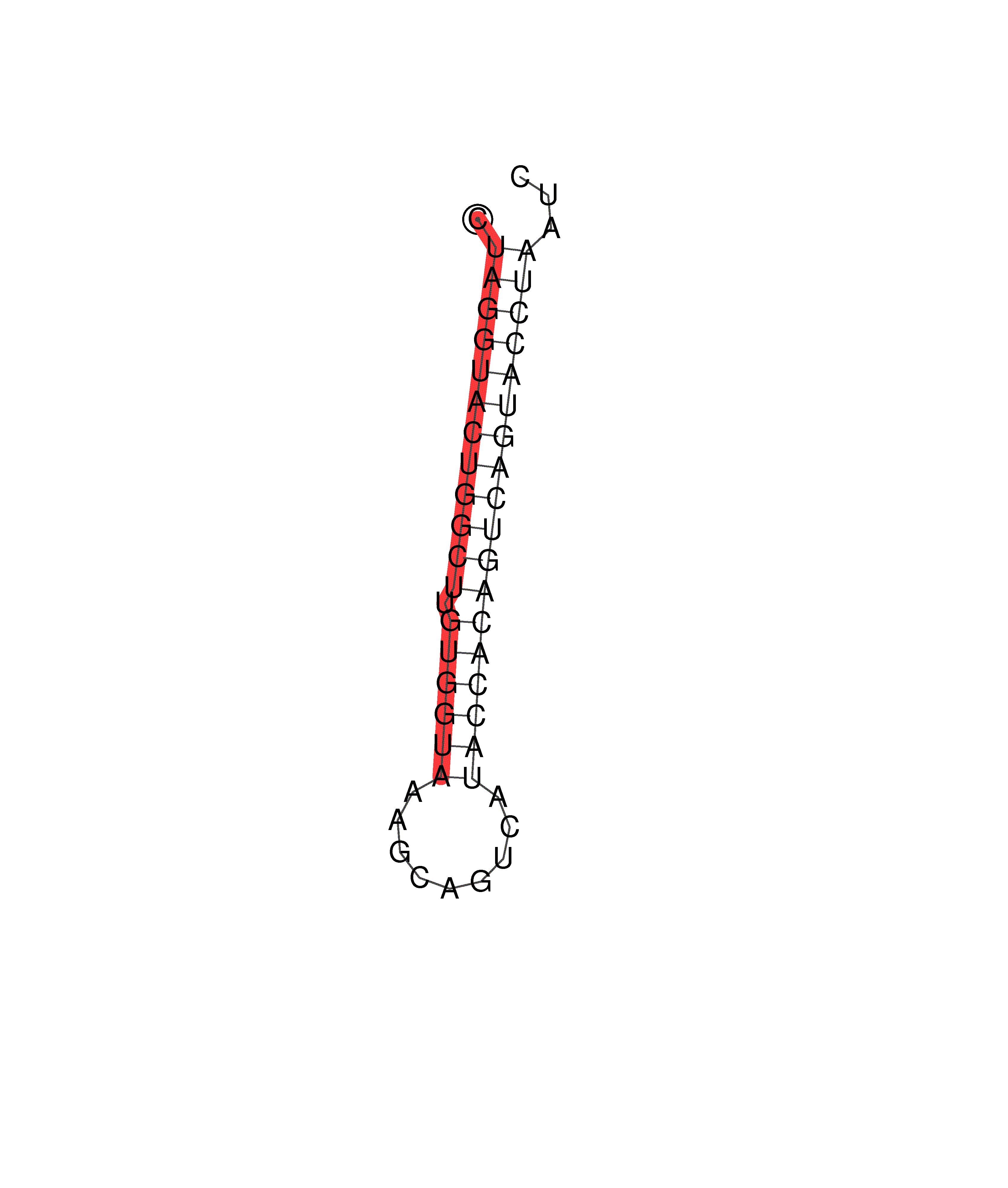


Y Fig. Secondary structure for novel_33

Z Fig.
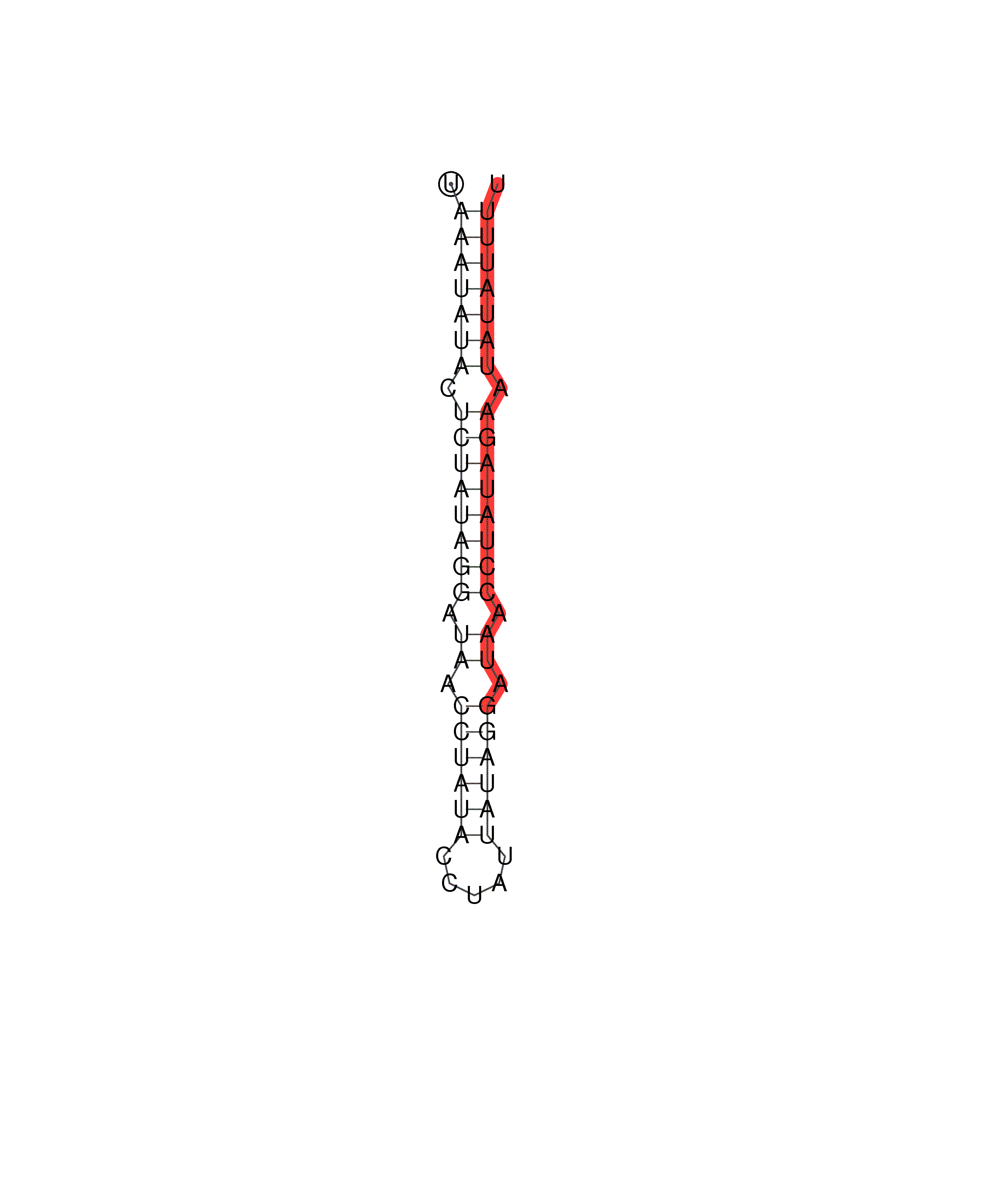
Secondary structure for novel_37


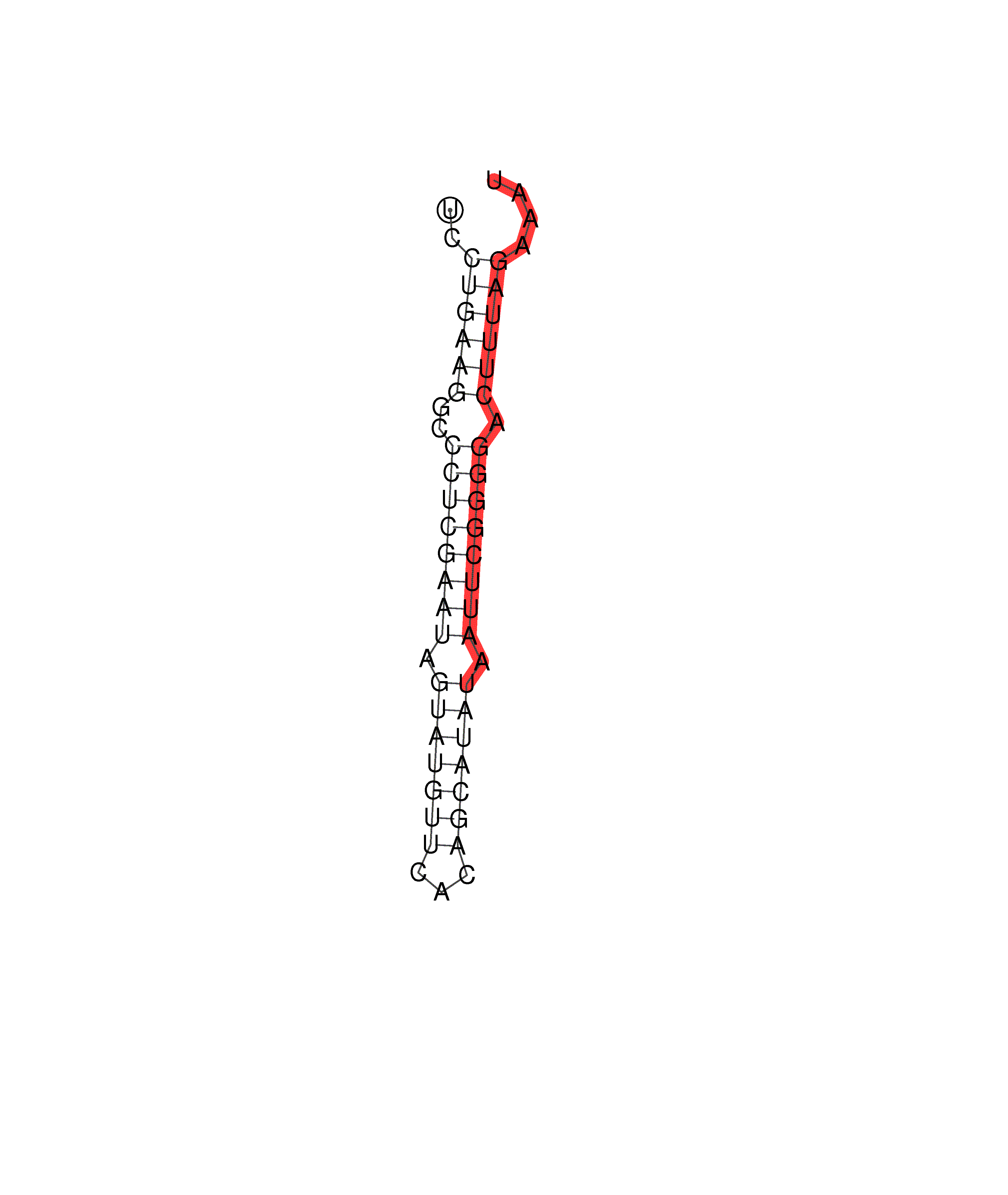


Aa Fig. Secondary structure for novel_38

Bb Fig.
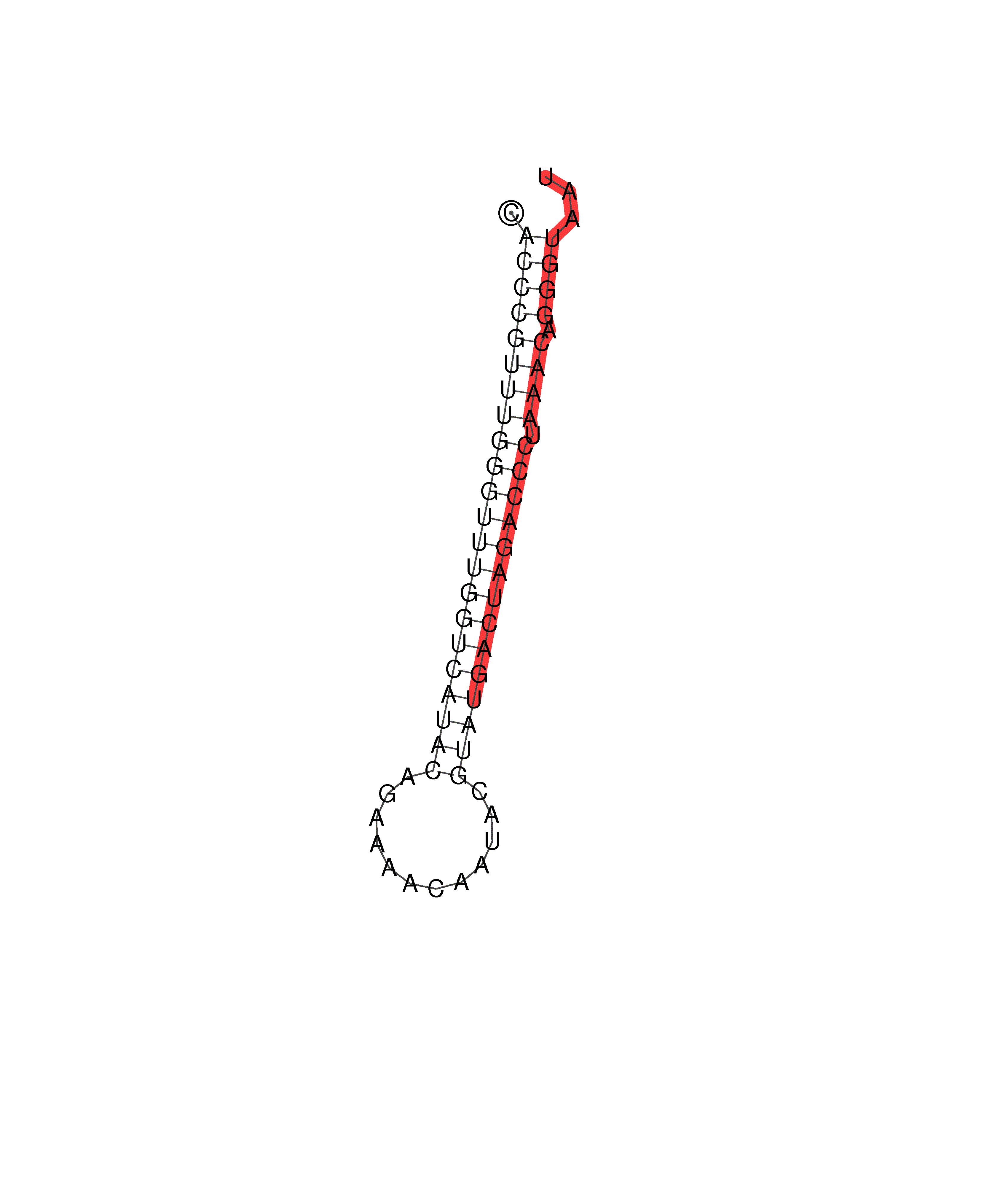
Secondary structure for novel_39


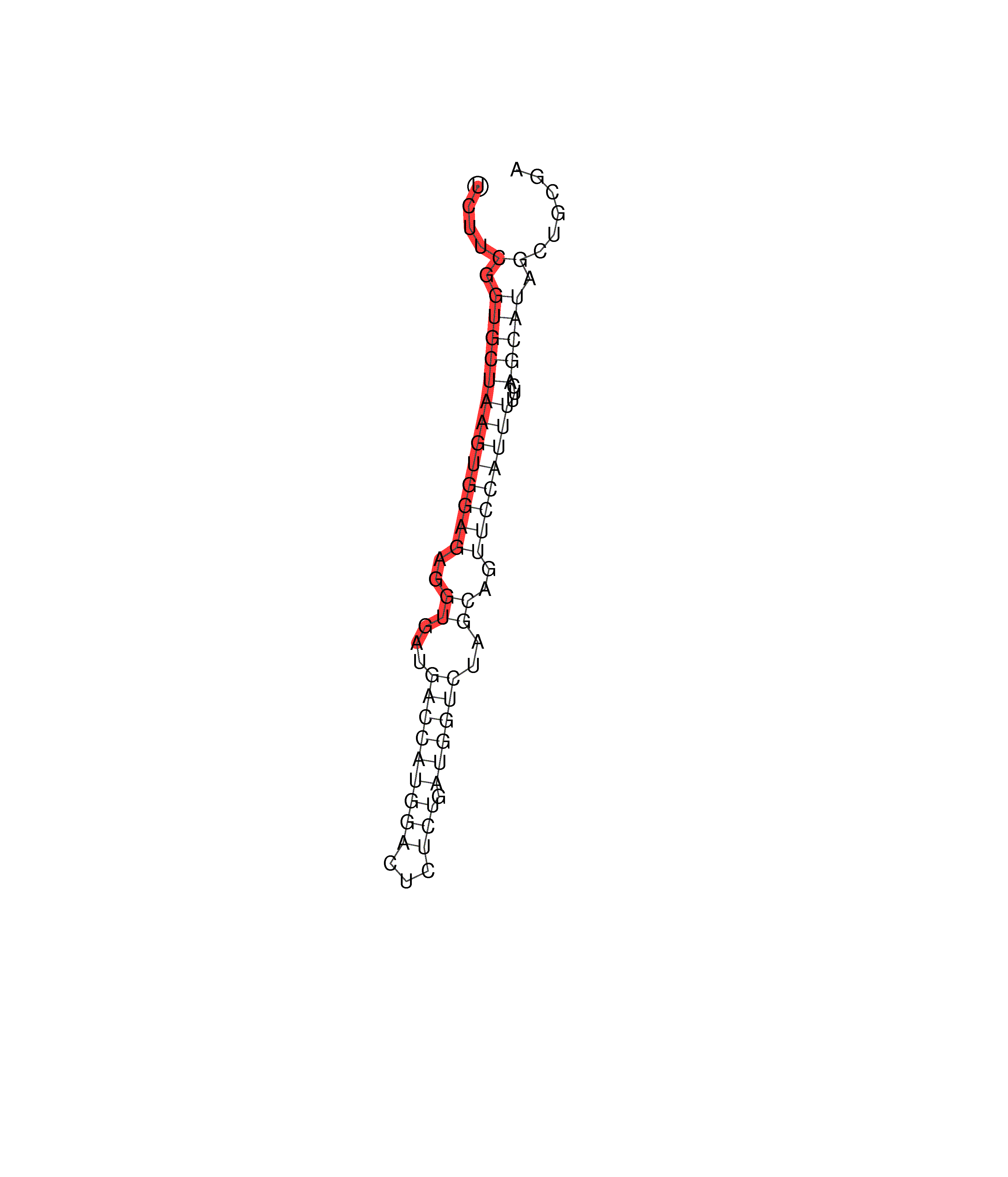


Cc Fig. Secondary structure for novel_40

Dd Fig.
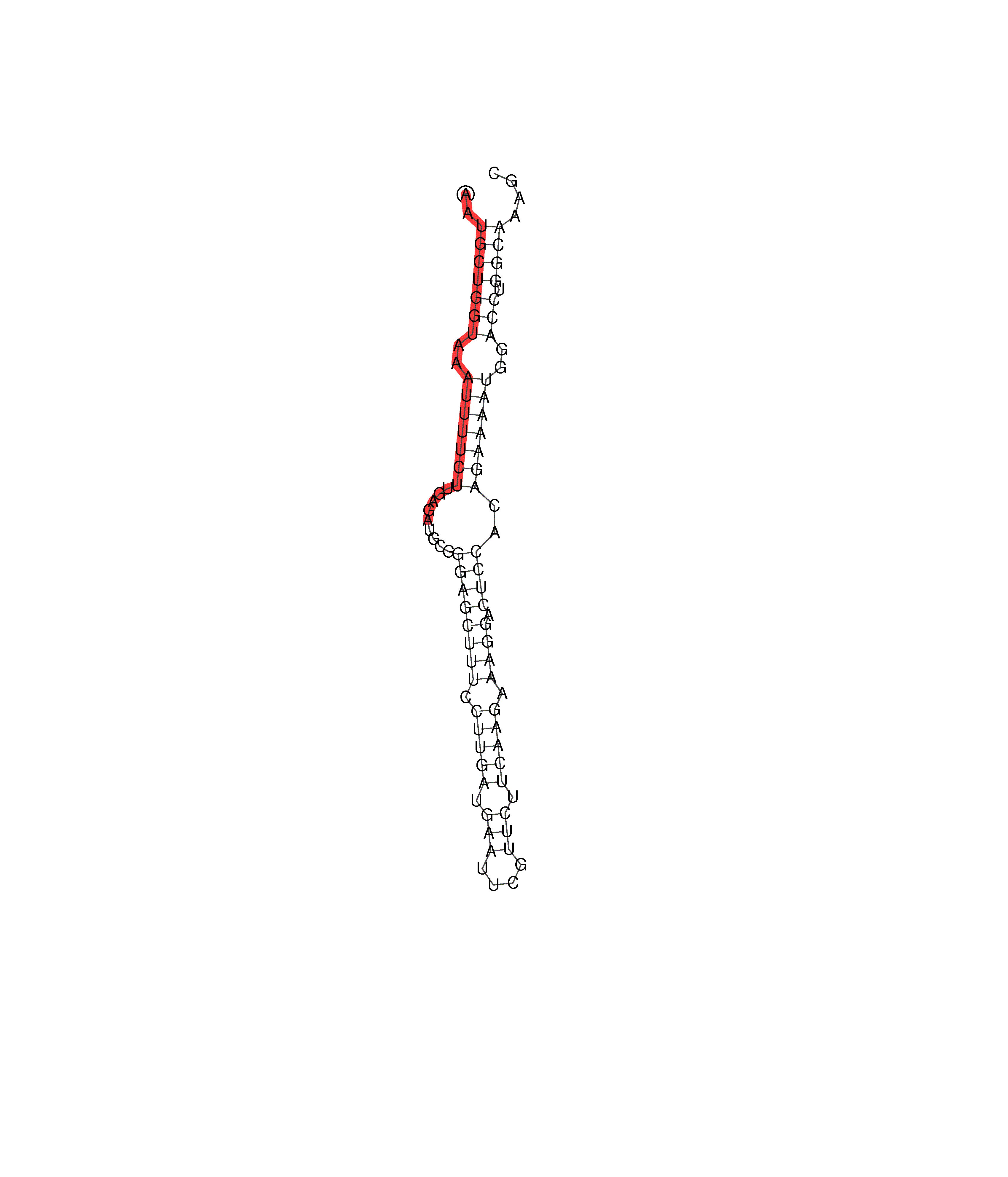
Secondary structure for novel_41


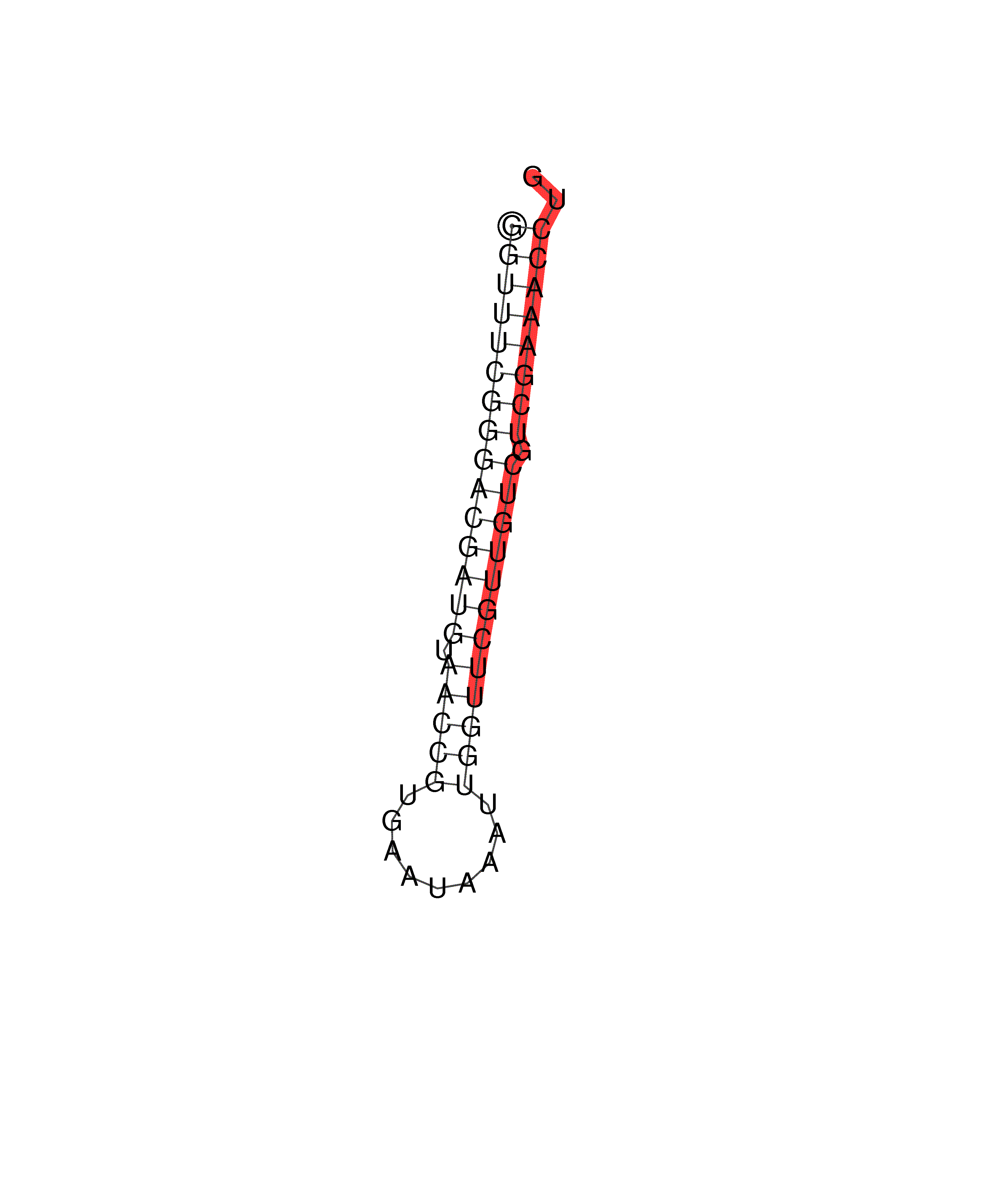


Ee Fig. Secondary structure for novel_42

Ff Fig.
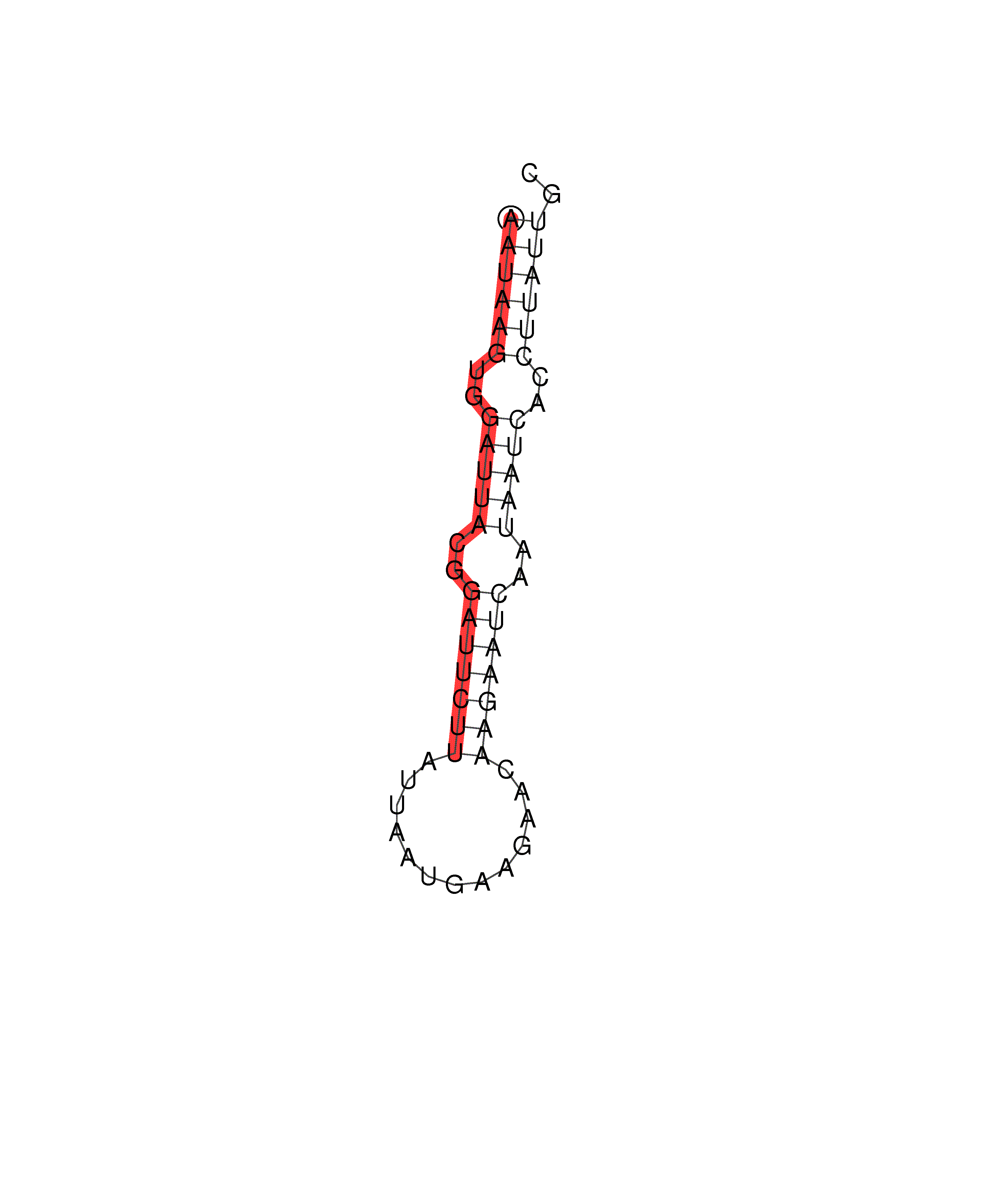
Secondary structure for novel_43

Gg Fig. Secondary structure for novel_44


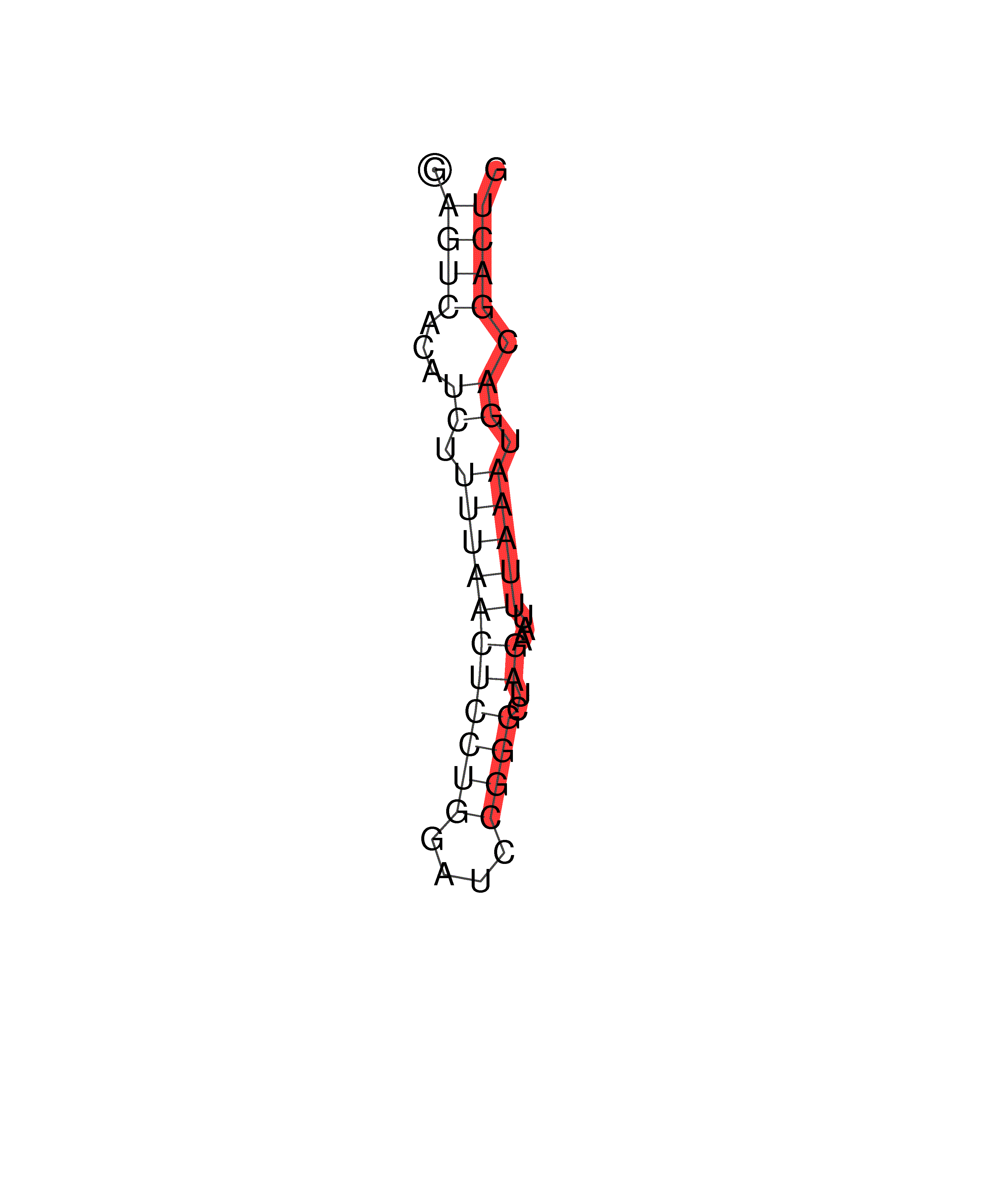


Hh Fig.
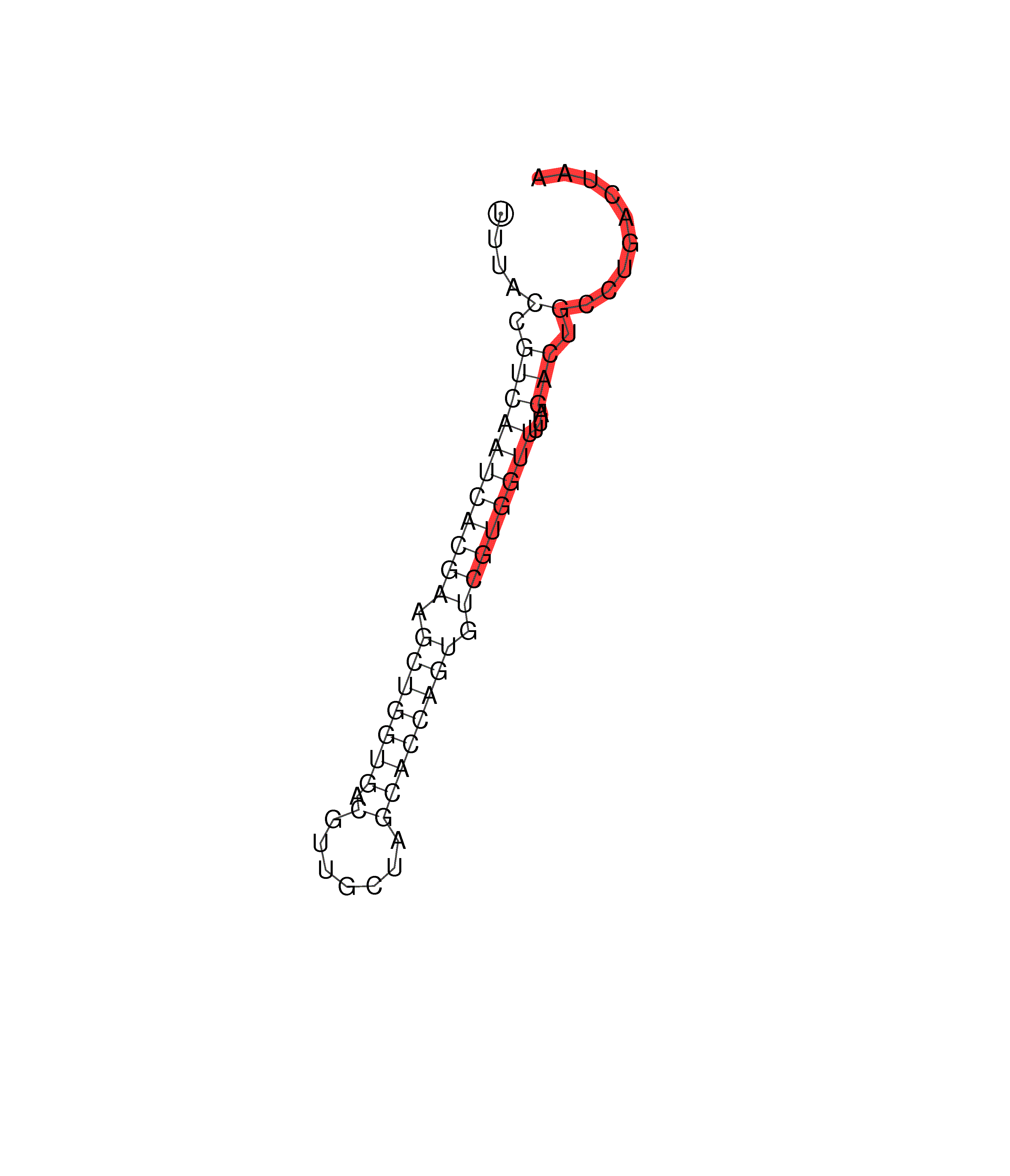
Secondary structure for novel_45


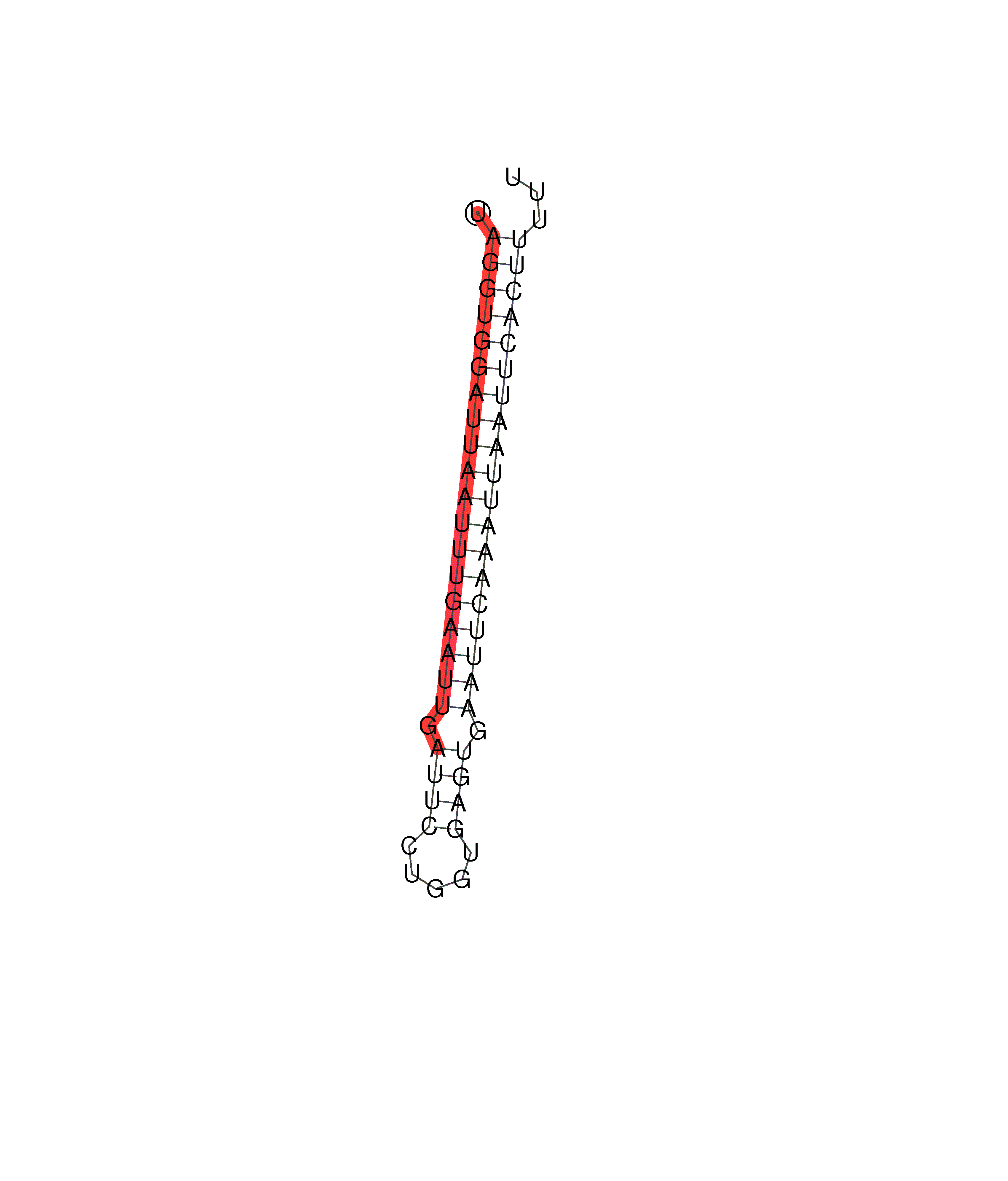


Ii Fig. Secondary structure for novel_46

Jj Fig.
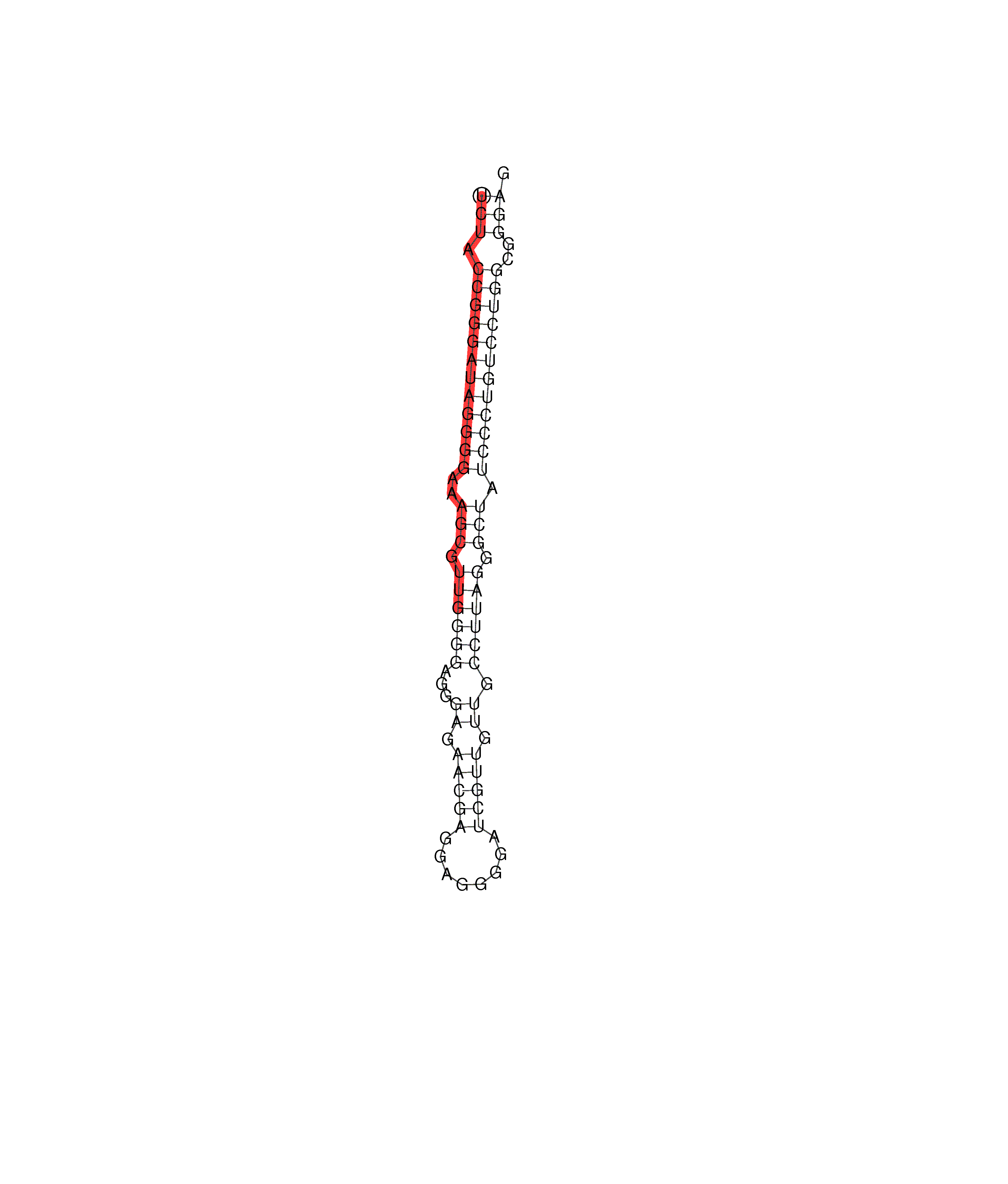
Secondary structure for novel_48

Kk Fig.
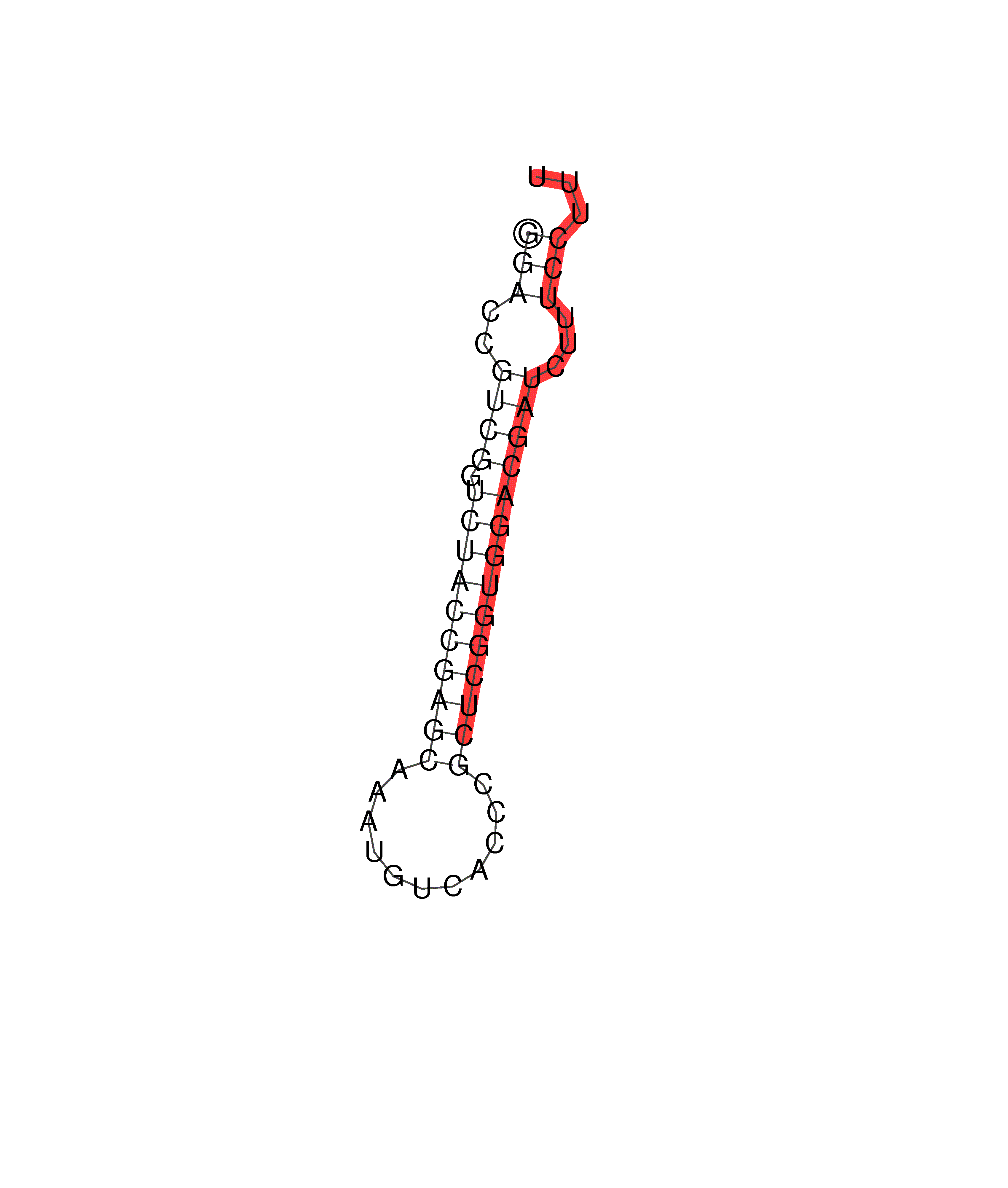
Secondary structure for novel_51

Ll Fig.
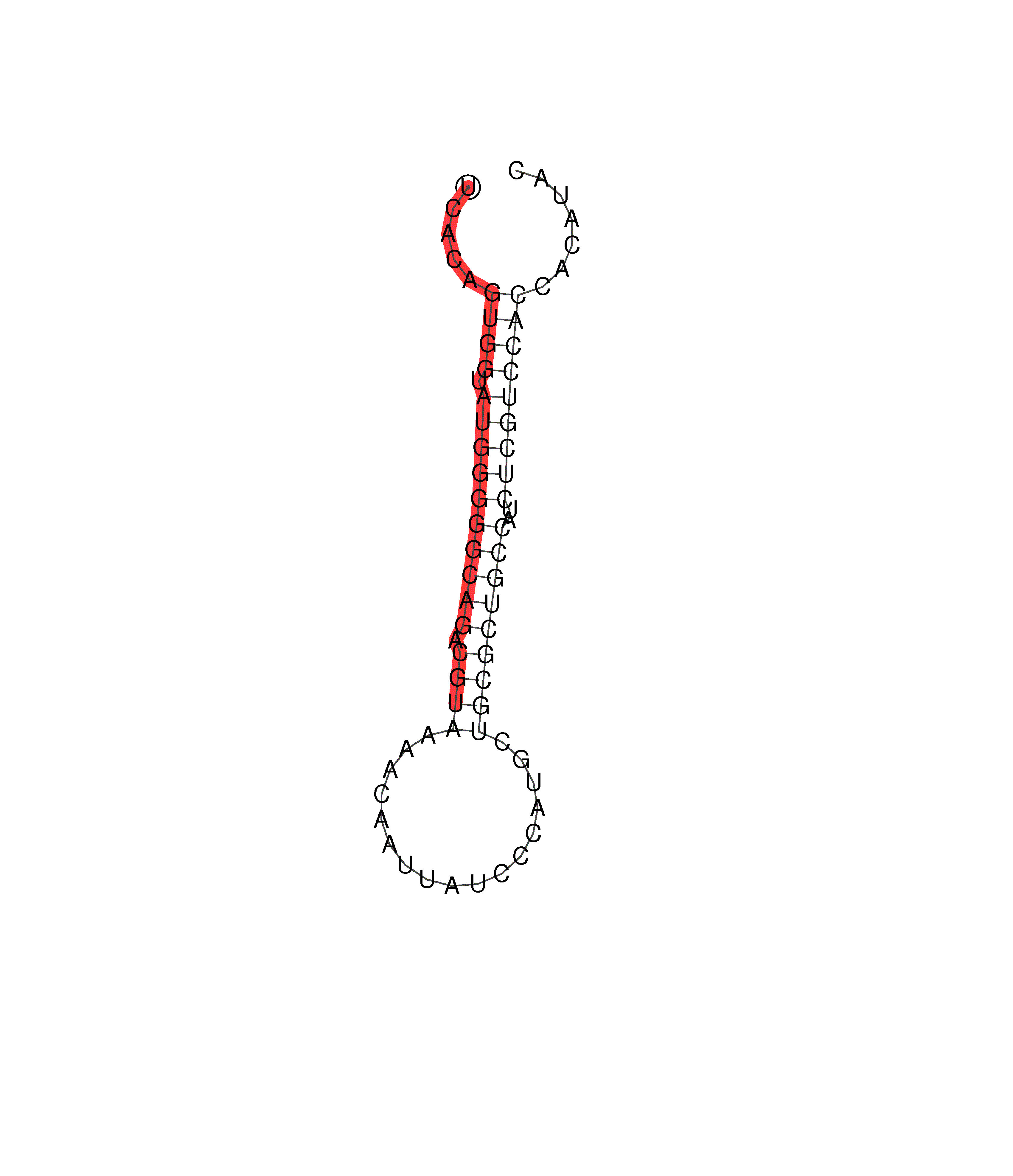
Secondary structure for novel_52


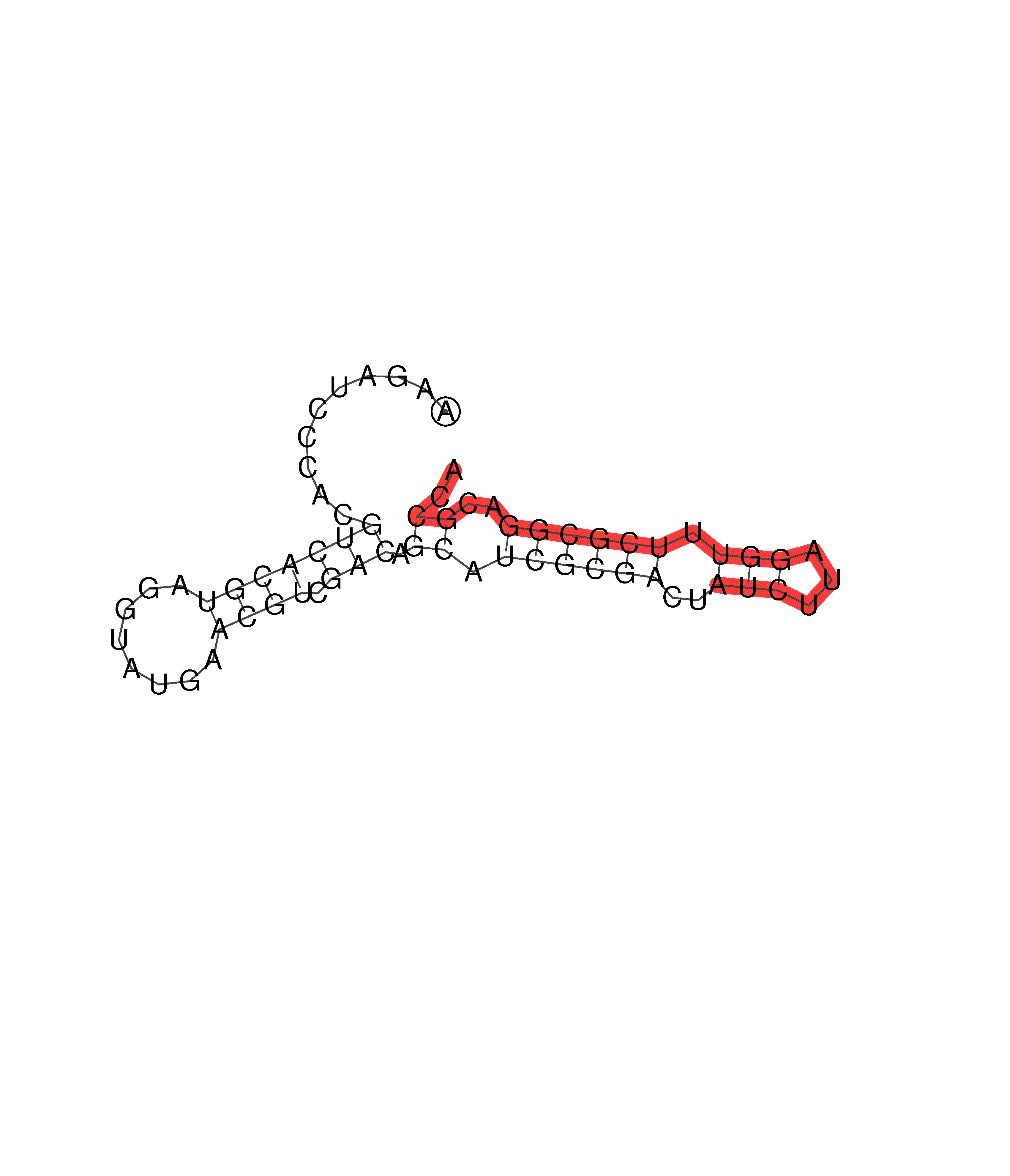


Mm Fig. Secondary structure for novel_53

Nn Fig.
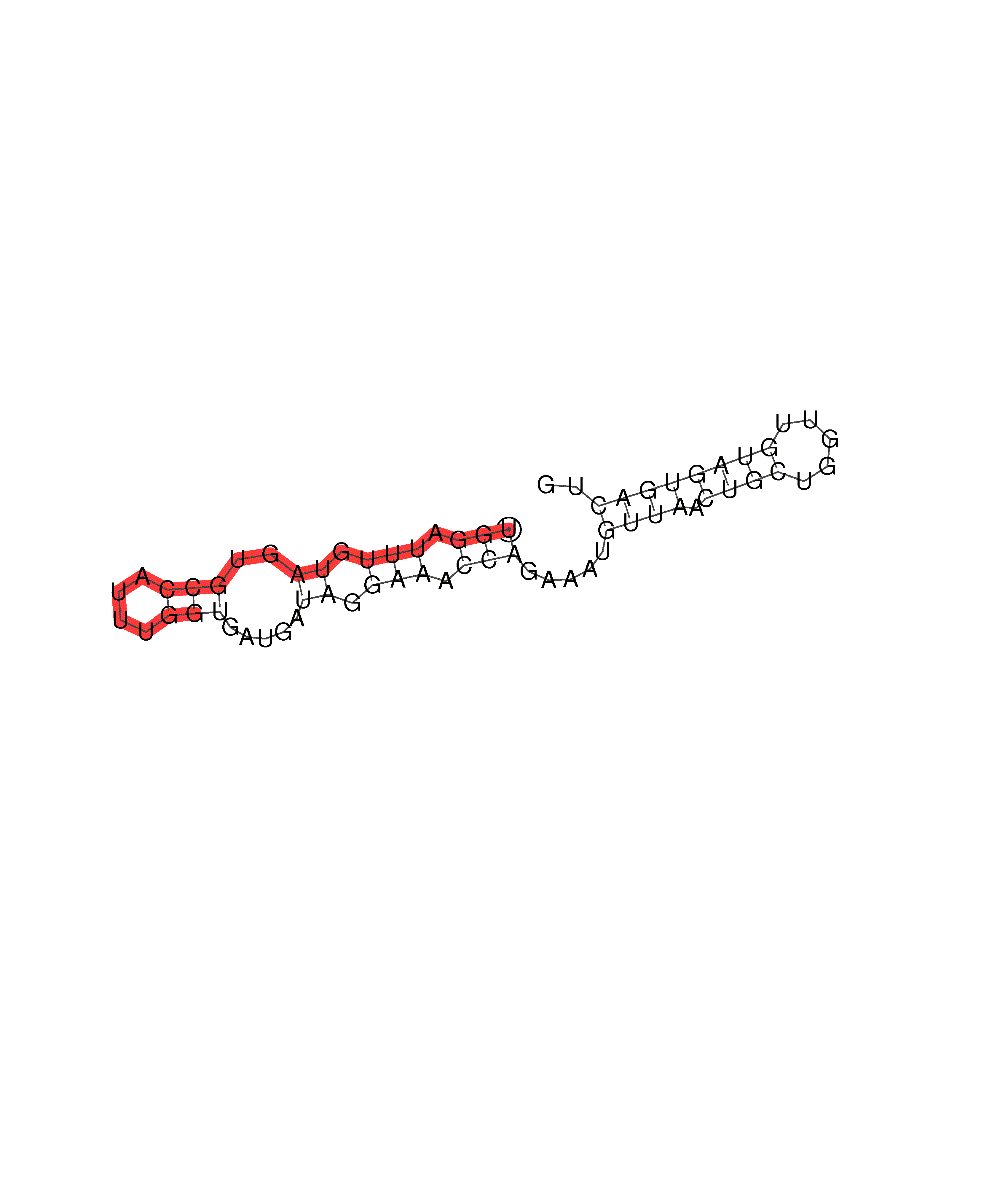
Secondary structure for novel_54


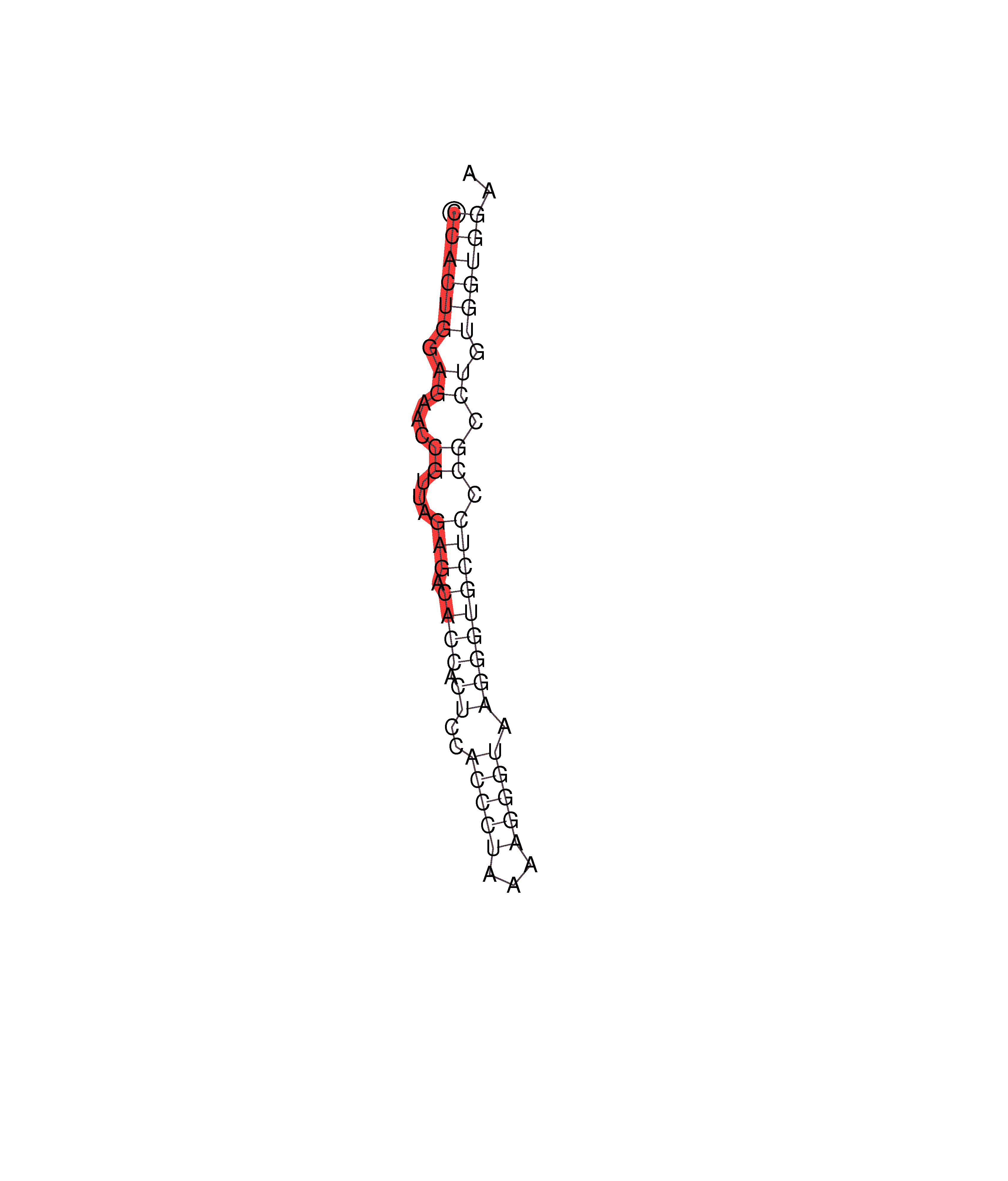


Oo Fig. Secondary structure for novel_55

Pp Fig.
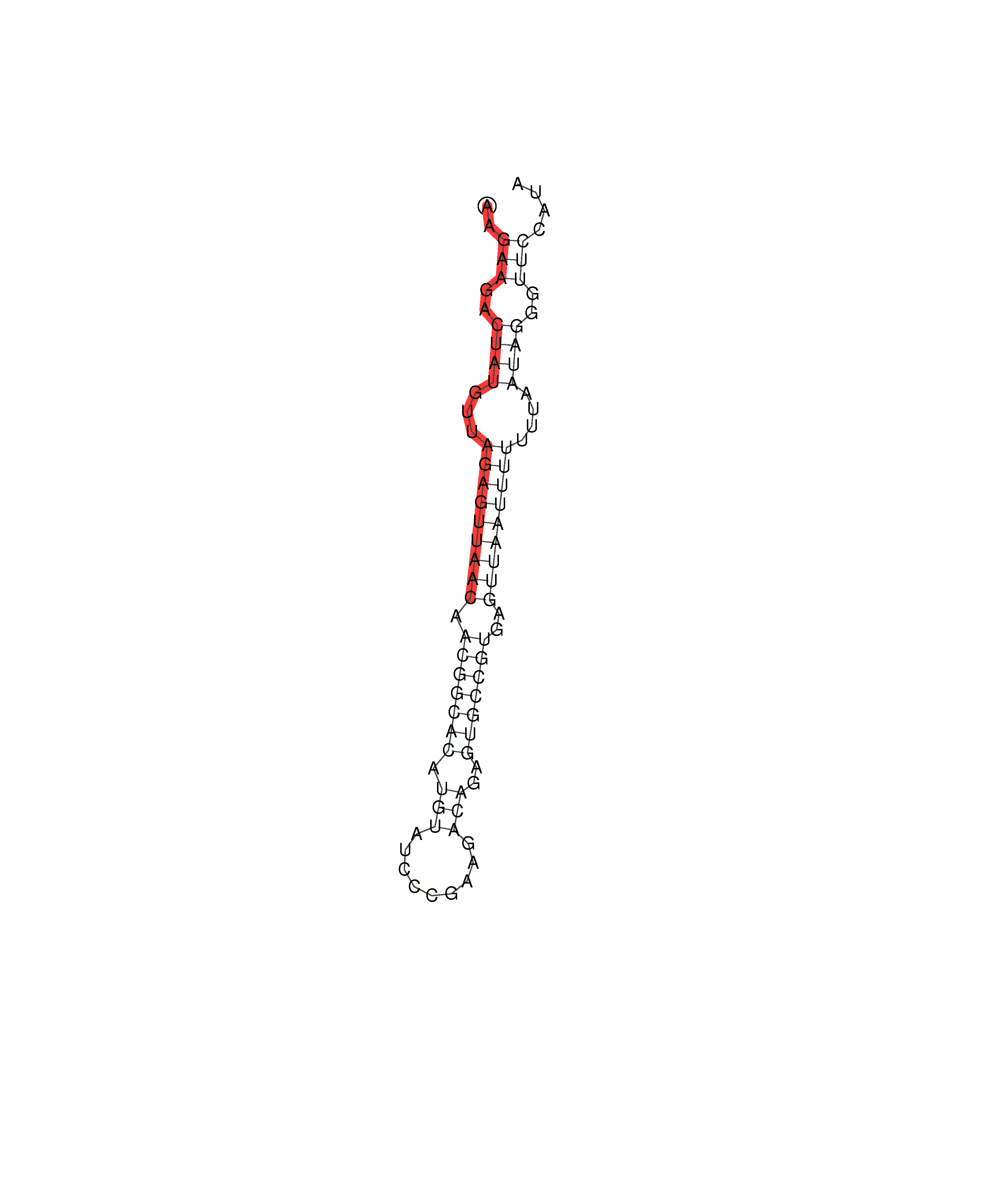
Secondary structure for novel_56


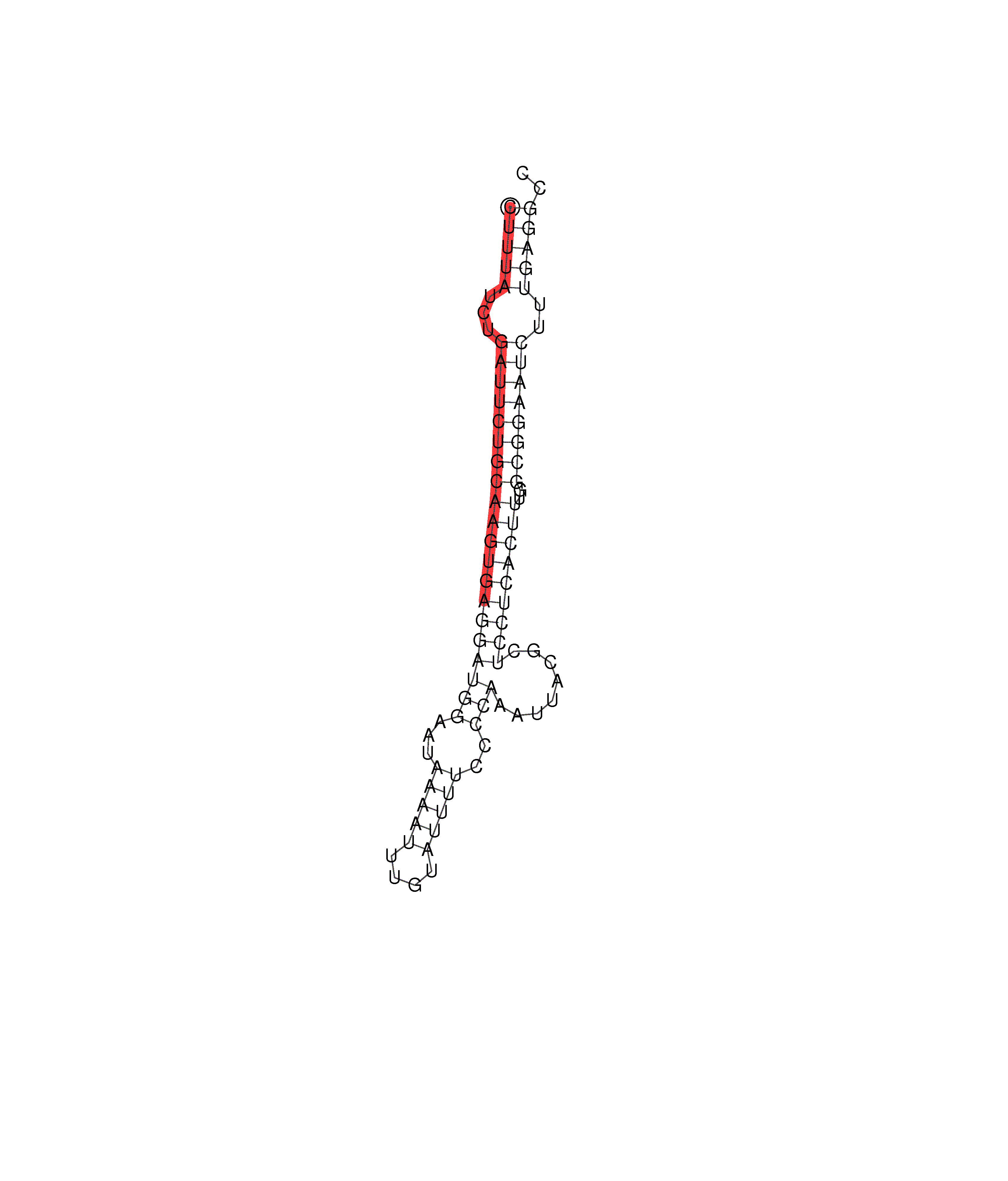


Qq Fig. Secondary structure for novel_57

Rr Fig. Secondary structure for novel_60


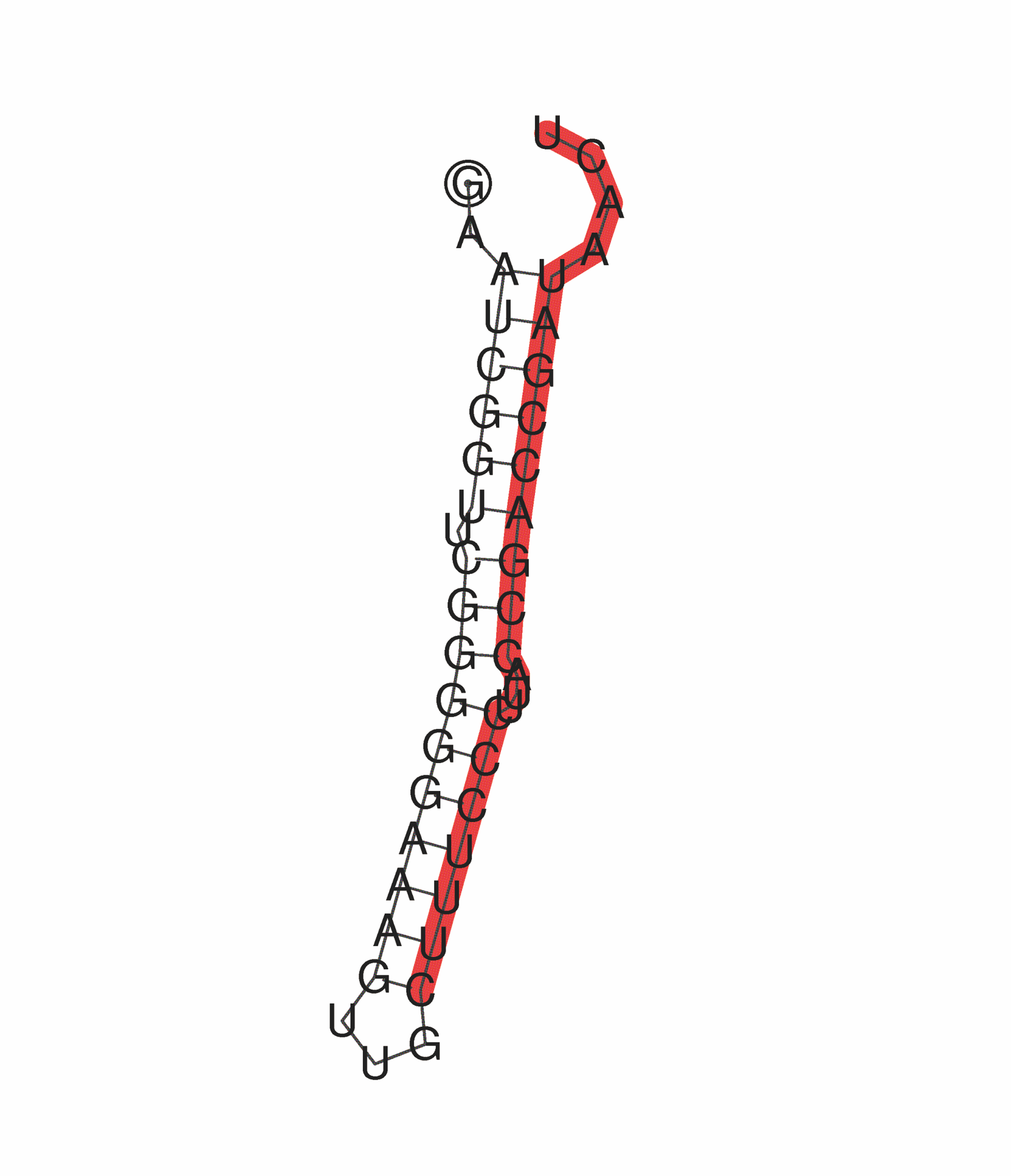

Supplement: S1 File — (DOC) [file pone.0180085.s007.doc]
